# The bidirectional interplay between RNA processing and mechanotransduction

**DOI:** 10.1016/j.celrep.2025.116175

**Published:** 2025-08-29

**Authors:** Gabrielle B. Bais, Jimena Giudice

**Affiliations:** 1 Curriculum in Cell Biology and Physiology, The University of North Carolina at Chapel Hill, Chapel Hill, NC, USA; 2 Department of Cell Biology and Physiology, The University of North Carolina at Chapel Hill, Chapel Hill, NC, USA; 3 Integrated Vascular Biology Training Program, The University of North Carolina at Chapel Hill, Chapel Hill, NC, USA; 4 McAllister Heart Institute, The University of North Carolina at Chapel Hill, Chapel Hill, NC, USA; 5 RNA Discovery Center, The University of North Carolina at Chapel Hill, Chapel Hill, NC, USA

## Abstract

Through mechanotransduction, cells sense and respond to mechanical stimuli from their environment. A mechanical stimulus is first detected by a mechanosensor, then converted into a biochemical signal, which can ultimately control the expression of genes. RNA processing, which includes canonical and alternative splicing, 3^′^ end polyadenylation, and 5^′^ end capping, is a mechanism that fine-tunes gene expression regulation. Here, we provide an overview of recent studies revealing substantial links between mechanotransduction and RNA processing, with a focus on alternative splicing and polyadenylation. We first describe the molecular players that are known to mediate mechanotransduction. Then, we examine how mechanical forces inform the functions of RNA-binding proteins. We next summarize recent investigations demonstrating that genes encoding mechanosensory proteins are alternatively spliced and how alternative splicing might impact isoform functions. Last, we discuss the role of mechanical forces on alternative splicing and polyadenylation landscapes.

## INTRODUCTION

Cells sense and respond to various stimuli from their physical environment via mechanotransduction, wherein mechanical cues are converted into biochemical signals that regulate transcriptional and post-transcriptional programs. Mechanotransduction is essential to all tissues, but is particularly important in cardiac, skeletal, and smooth muscles as well as bone, cartilage, and blood vessels because these tissues are constantly under mechanical stress.^1^ Mechanical forces experienced by cells differ depending on cell type and function and their surrounding environment. Mechanical forces commonly experienced by cells include hydrostatic pressure, stretching, fluid shear stress, extracellular matrix (ECM) stiffness, and extracellular fluid viscosity.^2^ Muscle cells can generate mechanical forces via contraction at the sarcomere (cardiac and skeletal myocytes) or actomyosin filaments (smooth muscle cells).^3^ Cell proliferation and differentiation, as well as stem cell fate, are greatly impacted by environmental mechanical forces.^4–7^ More narrowly, ECM stiffness in different tissues can direct mesenchymal stem cell fate decisions toward neurons, myoblasts, or osteoblasts.^8^ ECM stiffness also impacts various cell behaviors, including cell spreading, migration, matrix secretion, differentiation, division, apoptosis, and morphogenesis.^9^ Importantly, dysregulation of ECM composition or its biophysical and biomechanical properties contribute to cancer progression.^10–13^

**Figure 1.**
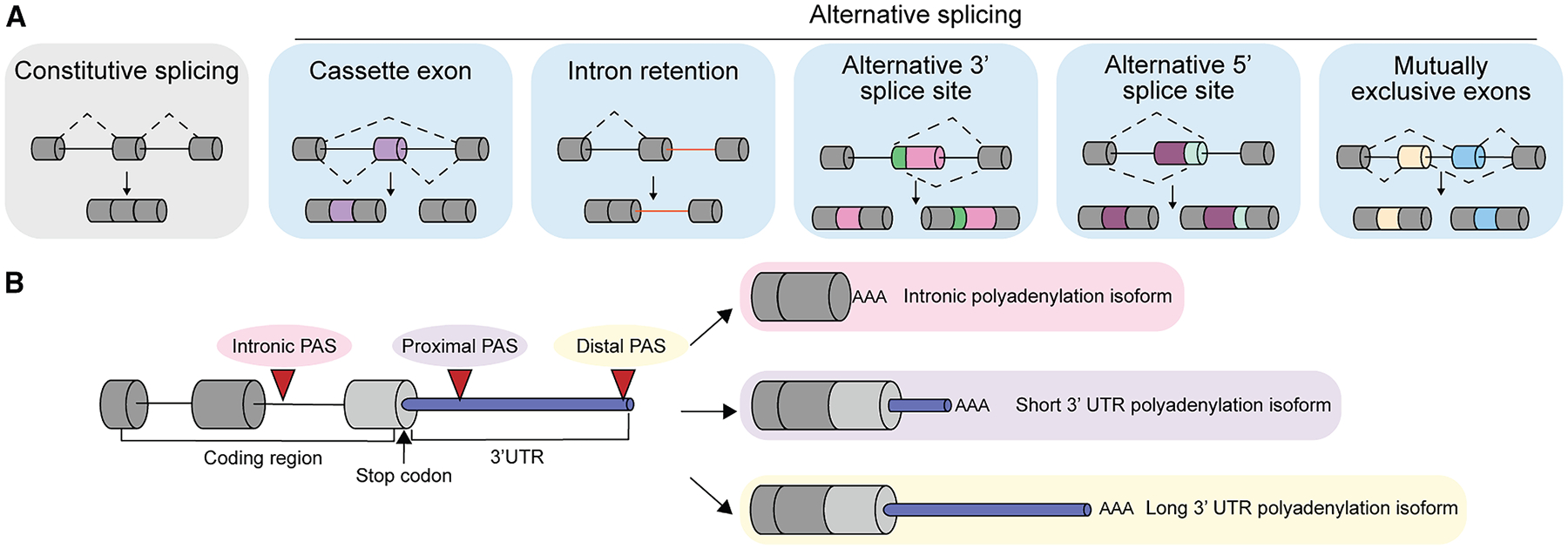
RNA processing as a regulator of gene expression (A) Alternative splicing is an RNA processing mechanism wherein the inclusion or exclusion of alternative regions results in different processed RNA transcripts. Constitutive splicing refers to the general inclusion of exons and splicing out of introns during RNA processing (left). The most common types of alternative splicing events are as follows: (1) cassette exons that can be either included or skipped, (2) intronic regions that instead of being spliced out are retained completely or partially, (3) alternative usage of 3^′^ or 5^′^ splice sites present within an exon, and (4) mutually exclusive exons that can be included or skipped but never at the same time (i.e., when one of them is included, the other one is skipped and vice versa). Constitutive exons are represented in gray, introns are represented as black lines, while alternatively spliced regions are in color. (B) Alternative polyadenylation is an RNA processing mechanism that generates RNA transcript diversity by using different polyadenylation sites (PASs) where mRNA cleavage and attachment of the poly(A) tail occur. PASs that are sometimes found within introns are known as intronic PASs. When an intronic PAS is upstream of a stop codon, both the coding sequence and the 3^′^ UTR are impacted. Cleavage and alternative polyadenylation also occur within the 3^′^ UTR, where there can be either a proximal PAS (relatively close to the stop codon) or a distal PAS (relatively far from the stop codon). Proximal PAS usage results in a shorter 3^′^ UTR than distal PAS usage.

Global changes in gene expression drive cellular adaptations to mechanical stimuli.^14–16^ Gene expression is regulated by a combination of chromosomal, transcriptional, and post-transcriptional subcellular events.^17–19^ Most protein-coding messenger RNAs (mRNAs) are co- and/or post-transcriptionally processed via constitutive and alternative splicing, 3^′^ end polyadenylation, and 5^′^ end capping, ultimately giving rise to mature mRNAs. RNA processing allows cells to fine-tune gene expression and protein production.^17,20^ Alternative splicing is an RNA processing mechanism wherein a single gene produces multiple mRNA transcripts and potentially different protein isoforms by including or skipping specific regions of the pre-mRNA. Constitutive splicing refers to the general inclusion of exons and splicing out of introns during RNA processing (Figure 1A, left). When genes are alternatively spliced, specific regions are selectively included or skipped in the mature transcript. The most common types of alternative splicing events are as follows: (1) cassette exons that can be either included or skipped, (2) intron retention events wherein intronic regions that instead of being spliced out are retained completely or partially, (3) alternative usage of 3^′^ and 5^′^ splice sites that can be present within an exon, and (4) mutually exclusive exons which are two adjacent alternatively spliced exons that can be included or skipped but never at the same time (Figure 1A). Alternative polyadenylation occurs when multiple polyadenylation sites (PASs) exist in a transcript, allowing the generation of 3^′^ untranslated regions (UTRs) of different lengths (Figure 1B). Alternative splicing and alternative polyadenylation greatly expand proteomic diversity and protein function in cells^21–24^ and play key roles in a wide variety of pathophysiological states.^24,25^ Alternative splicing and alternative polyadenylation can be connected or interdependent.^20^ In mammals, the last intronic sequence on the 3^′^ end of an mRNA transcript (terminal intron) is often spliced out after the addition of a polyadenylic acid (poly[A]) tail.^20,26^ Newer research suggests that splicing of the terminal intron may be dependent on poly(A) tail length.^20,27^ Moreover, intron retention in general can occur due to the presence of intronic PASs.^20,21^

Both RNA processing and mechanotransduction are implicated in development and disease.^1,2,28–30^ Here, we will discuss in depth the growing body of research revealing how RNA processing and mechanotransduction act synchronously to modulate cellular adaptations. In other words, we will review how mechanotransduction regulates or is regulated by RNA processing in physiology and disease. First, we will provide a brief overview of the canonical players and pathways involved in mechanotransduction. Second, we will describe how RNA-binding proteins (RBPs) function as molecular drivers of RNA processing during mechanotransduction. Third, we will discuss how alternative splicing influences the functions of various molecules that start the mechanotransduction cascade at the plasma membrane and the ECM, including fibronectin, vinculin, Piezo1 and Piezo2, the potassium two-pore domain channel subfamily K member 2 (also known as Tandem of p domains in a Weak Inward rectifying K^+^ channel [TWIK]-related K^+^ channel or TREK-1), and multiple components of the hair cell mechanotransduction complex. Lastly, we will summarize the research demonstrating that mechanical forces induce global transitions in alternative splicing and alternative polyadenylation landscapes.

## MECHANOTRANSDUCTION IS REGULATED BY NUMEROUS CELLULAR COMPONENTS

**Figure 2.**
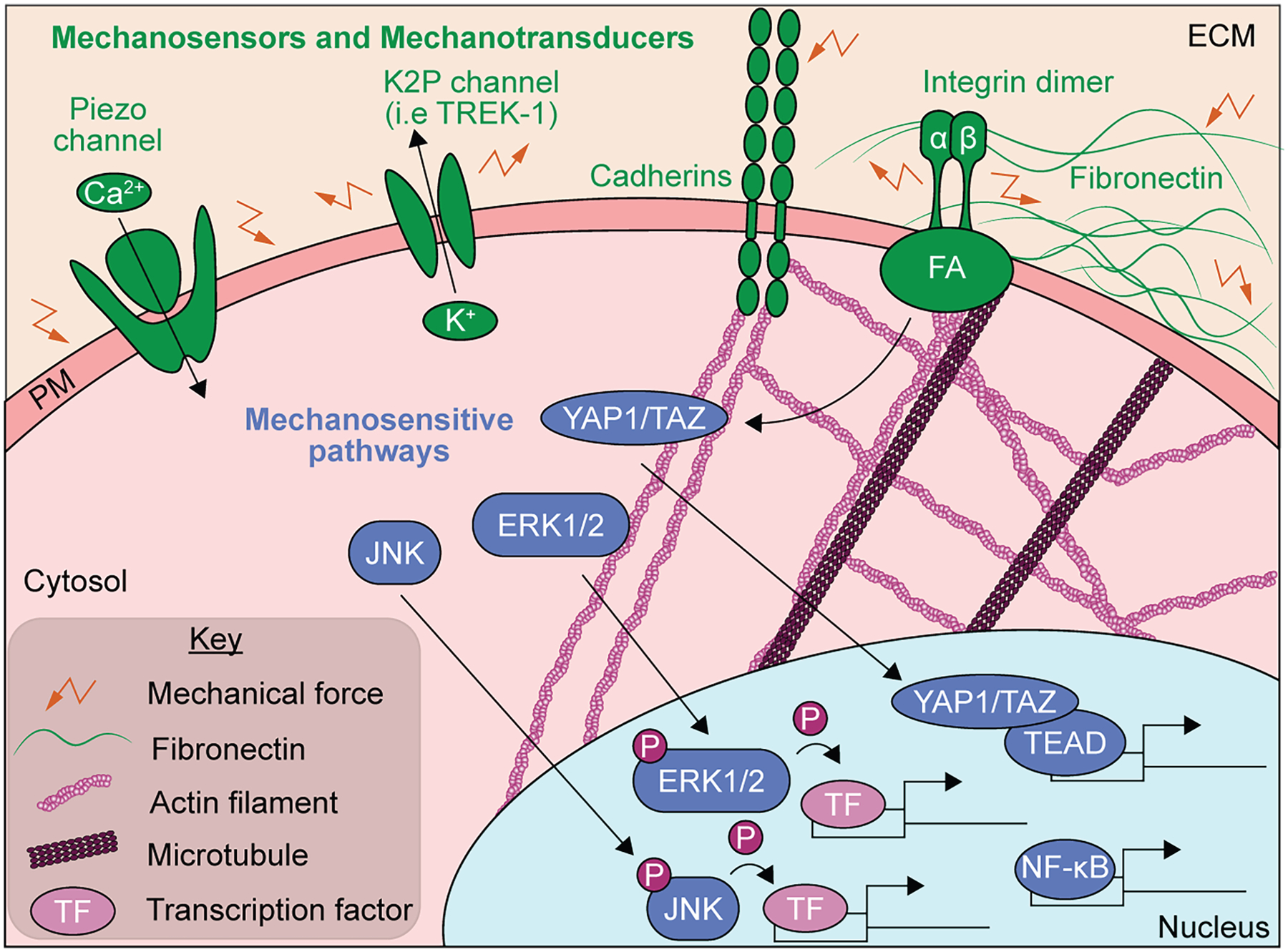
Players involved in each step of cellular mechanotransduction Mechanical forces can be intercepted by a variety of mechanosensors and mechanotransducers (green). Piezo channels and two-pore domain potassium (K2P) channels (such as TREK-1) are ion channels that are activated by mechanical force. When activated, Piezo channels allow for calcium ions (Ca^2+^ to flow into cells, while activated K2P channels transport potassium ions (K^+^) out of cells (top left). The cell adhesion molecules, integrins and cadherins, also function as mechanosensors capable of transmitting mechanical signals into the cell via the actin cytoskeleton, though microtubules have also been implicated in integrin mechanotransduction (top middle). Mechanotransduction initiated by integrin receptors is propagated by focal adhesion (FA) complexes. Furthermore, the ECM component fibronectin acts as a mechanosensor due to its ability to undergo mechanically regulated conformational changes, which in turn trigger integrin signaling (top right). After a mechanical stimulus is intercepted by a mechanosensor, several intracellular mechanosensitive signaling pathways (blue) are activated downstream (mechanotransduction). Integrin receptors and FA molecules participate in the Hippo signaling pathway, which ultimately leads to the activation of the co-transcriptional activator molecules YAP1 and TAZ (YAP1/TAZ complex). When activated, YAP1/TAZ complexes translocate from the cytosol to the nucleus, where they interact with TEAD transcription factors to drive changes in gene expression. NF-κB activation and regulation of transcription also respond to mechanical forces. Further, the MAPKs extracellular signal-regulated kinase (ERK) and c-jun N-terminal kinase (JNK) are both activated by mechanical forces. Upon activation, ERK and JNK are shuttled from the cytosol to the nucleus, where they phosphorylate and activate target transcription factors, which in turn drive changes in gene expression.

Mechanotransduction encompasses the sensing of mechanical stimuli, the triggered signal transduction,and the subsequent cellular response to these stimuli. Several protein complexes and signaling pathways are implicated at specific stages of mechanotransduction (Figure 2). Disruption of mechanotransduction results in alterations in organism development, cardiomyopathies, hearing loss, bone and cartilage disorders, axial myopia, glaucoma, arteriosclerosis, and cancer.^1,2^ Mechanotransduction begins with the cellular sensation of mechanical forces, which requires the action of mechanotransduction mediators that can function as mechanosensors and/or mechanotransducers.^1,31^ Mechanosensors refer to proteins that, via mechanically induced conformational changes or modifications, sense mechanical cues from the surrounding environment or cells. Mechanotransducers refer to proteins that transmit and convert the mechanical stimulus into a biochemical signal.^32,33^ Often, mechanotransducers are at least at some level also mechanosensors.

Mechanically gated and stretch-activated ion channels, such as Piezo proteins^34^ and two-pore domain potassium channels,^35^ are important for mechanotransduction in multiple tissues and cell types (Figure 2, top left). In other cases, mechanosensors and mechanotransducers are cell-type specific, as is the case for the glycocalyx, a matrix of glycoproteins and proteoglycans on the apical side of the endothelium^36^ and the mechanotransduction complex in sensory hair cells.^37^ The function of the glycocalyx is to sense changes in sheer stress on blood vessels generated by blood flow.^36,38^ Cell-cell junctional receptors and cell adhesion molecules such as integrins^39,40^ and cadherins^41^ are critical mechanotransducers across cell types (Figure 2, top right). Further, force-induced conformational changes in ECM proteins like fibronectin can activate integrin signaling in cells^9,41–43^ (Figure 2, top right). Similarly, cytoskeleton tension or compression can activate mechanosensitive pathways.^44,45^ The mitogen-activated protein kinase (MAPK),^46^ the nuclear factor-kappa B (NF-κB),^47,48^ and the Hippo^49,50^ pathways are all particularly mechanosensitive (Figure 2, bottom in blue) and often trigger transcriptional changes in response to mechanical stimuli.^14,15,51–53^

Another key layer of gene regulation via mechanotransduction is through the linker of nucleoskeleton and cytoskeleton complex and the nuclear lamina, wherein both tether the nucleus to the cytoskeleton.^54,55^ Changes in gene expression are in part driven by the direct anchoring of actin caps to the nucleus.^56,57^ Mechanical stretching of chromatin can also induce transcriptional responses in cells.^54,55^ In this manner, the nucleus, which controls the structure and composition of the nuclear envelope, chromatin organization, and gene expression, is important for mechanotransduction.^58^

## RBPs CAN REGULATE RNA PROCESSING DURING MECHANOTRANSDUCTION

RNA processing as well as all other aspects of RNA biology are highly regulated by RBPs.^59–61^ This section will discuss recent studies showing that the expression and/or activity of RBPs respond to mechanical stimuli, as well as examples of RBP expression and/or activity impacted by mechanotransduction.

### HNRNPC activity is regulated by changes in mechanical forces during heart failure

**Figure 3.**
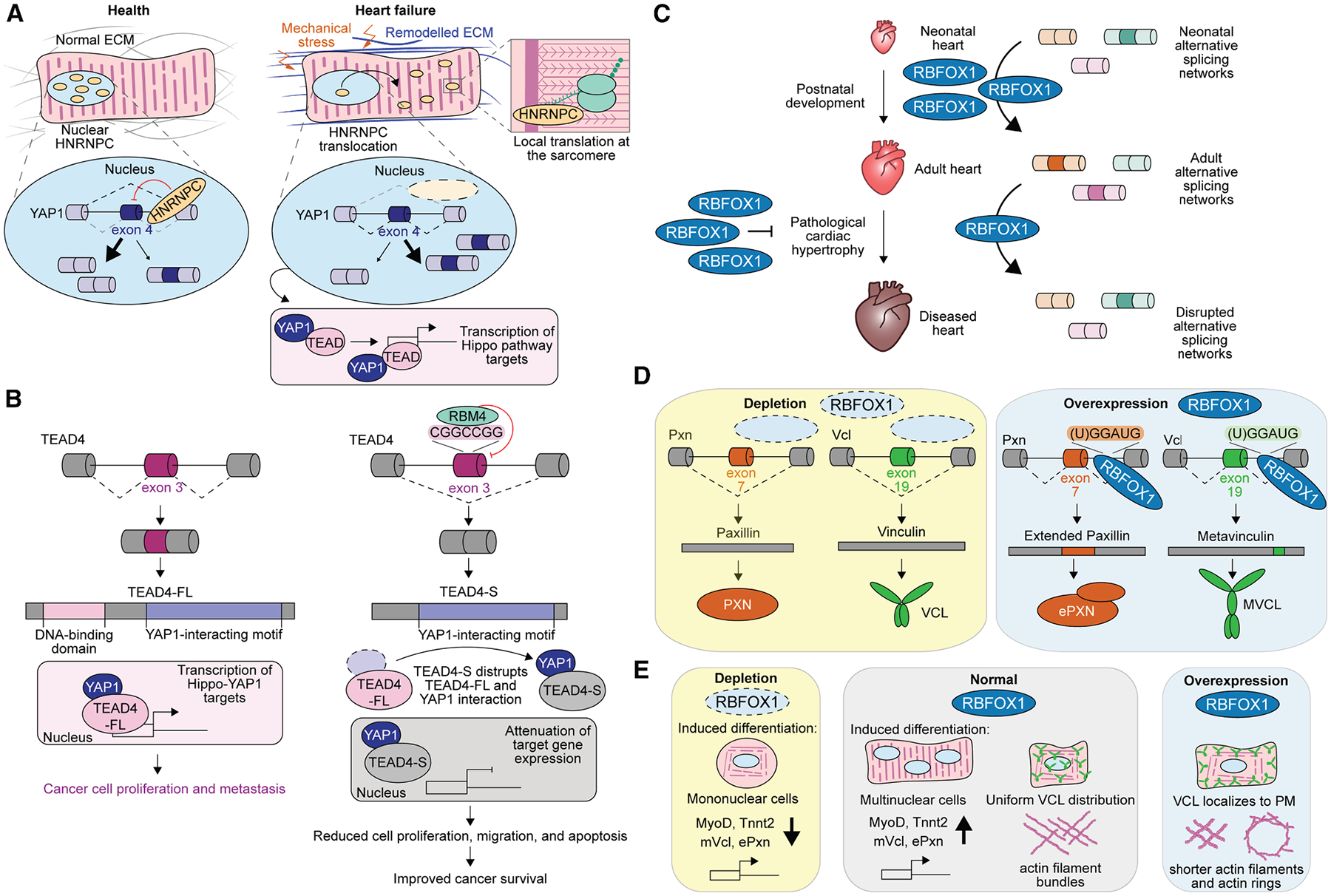
Mechanical forces influence the activity of RBPs (A) In healthy cardiomyocytes, HNRNPC localizes to the nucleus where it promotes skipping of YAP1 exon 4 (left). During heart failure, the ECM undergoes remodeling, and as a result, HNRNPC shuttles to the sarcomere, where it functions in local translation. Because there is less nuclear HNRNPC, YAP1 exon 4 inclusion is increased. When exon 4 is included, YAP1 readily activates TEAD transcription factors, which drive the transcription of Hippo pathway targets (right). This illustration is based on work published in Martino et al.^66^ (B) The transcription factor TEAD4 is alternatively spliced at exon 3. When exon 3 is included, the full-length TEAD4 (TEAD4-FL) is produced. TEAD4-FL contains the DNA-binding domain and YAP1-interacting motif. TEAD4-FL can bind YAP1 and drive transcription of Hippo-YAP1 targets. In cancer, the inclusion of TEAD4 exon 3 is associated with tumor cell proliferation and metastasis (bottom left). The RBP RBM4 binds a CGGCCGG motif within TEAD4 exon 3 and induces its skipping, which results in the production of a short TEAD4 protein isoform (TEAD4-S). Since exon 3 encodes the DNA-binding domain of TEAD4, TEAD4-S protein does not have a DNA-binding domain but retains the YAP1-interacting motif. Thus, TEAD4-S isoforms compete with TEAD4-FL for YAP1 binding. When YAP1 binds TEAD4-S, transcription is not activated, and there is an attenuation of transcription of Hippo-YAP1 targets. As a result, cancer cells expressing high levels of RBM4 exhibit reduced cell proliferation, migration, and apoptosis, and consequently, patients with higher levels of RBM4 have improved chances of cancer survival (right). This illustration is based on results published in Qi et al.^69^ (C) RBFOX1 is upregulated during postnatal heart development and is downregulated in pathological cardiac hypertrophy. Mice overexpressing RBFOX1 present reduced progression of pathological cardiac hypertrophy due to RBFOX1 regulation of alternative splicing. This illustration is based on findings from Gao et al.^70^ (D) RBFOX1 regulates alternative splicing of the pre-mRNAs of two focal adhesion proteins, paxillin (PXN) and vinculin (VCL). When RBFOX1 is depleted, Pxn exon 6 and Vcl exon 19 are skipped, generating PXN and VCL proteins (left), whereas when RBFOX1 is overexpressed, Pxn exon 6 and Vcl exon 19 are included via RBFOX1 binding to a (U)GGAUG motif in the intron downstream of these alternative exons. These alternative splicing events result in the production of extended PXN (ePXN) and metavinculin (MVCL) proteins (right). (E) In cultured H9C2 embryonic cardiomyocytes, the abundance of RBFOX1 regulates cellular morphology, gene expression, and actin cytoskeleton. When RBFOX1 is depleted (left), differentiated cardiomyocytes were more frequently mononuclear when compared to cells expressing endogenous RBFOX1, which became more often multinucleated after differentiation (middle). Moreover, differentiated RBFOX1-depleted cells exhibited reduced expression of myocyte differentiation markers such as myoblast determination protein 1 (MyoD) and cardiac troponin T (Tnnt2). MVcl and ePxn expression was also downregulated at the mRNA level in RBFOX1-depleted cardiomyocytes when compared to differentiating cells with normal RBFOX1 levels. RBFOX1 overexpressing cells (right) were overall more elongated, and VCL was increasingly localized to the plasma membrane (PM) when compared to wild-type cardiomyocytes (middle). Moreover, RBFOX1 overexpressing cells contained notably shortened actin filaments and actin rings when compared to wild-type. The illustrations in (D) and (E) are based on Zorn et al.^71^

The composition and stiffness of the ECM greatly contribute to the activation of mechanotransduction pathways,^9^ and this is especially important in tissues like the heart, where failures in sensing and transducing mechanical cues can lead to fatal diseases. To identify commonly upregulated genes controlling ECM remodeling in heart disease, Martino and colleagues performed *in silico* analysis of publicly available transcriptional profile arrays from ischemic and non-ischemic heart diseases in murine models and heart transplant patient data.^62–65^ A common trend noted by Martino et al. was that genes encoding proteins with RNA-binding domains were upregulated in cardiac pathologies compared to healthy individuals,^66^ suggesting that RBPs are involved in the phenotypic alterations occurring in heart diseases regardless of disease etiology. While RBP expression in general was upregulated in cardiac disease, the only RBP to be upregulated across all the datasets^62–65^ was the heterogeneous nuclear ribonucleoprotein C (HNRNPC).^66^ HNRNPs contain a nuclear localization signal and tend to predominantly localize in the nucleus, though post-translational modifications or interactions with other RBPs can alter their subcellular localization and, as a consequence, their functions.^67,68^ In healthy cardiomyocytes, HNRNPC is localized to the nucleus where it regulates splicing (Figure 3A, left), but in heart failure, HNRNPC is shuttled to the sarcomere where it interacts with sarcomere RNAs and has been proposed to regulate local translation (Figure 3A, right).^66^ Adding to the connection between mechanotransduction and HNRNPC function, this RBP has been shown to increasingly shuttle to the sarcomere in response to cell spreading and cytoskeletal tension in human induced pluripotent stem cell-derived cardiomyocytes.^66^

Multiple HNRNPC splicing targets are involved in mechanotransduction pathways. One of these targets is the Yes-associated protein 1 (YAP1), which is a main effector of the mechano-sensitive Hippo cascade.^66^ Nuclear HNRNPC promotes the skipping of YAP1 exon 4 (Figure 3A, left). Mechanically induced shuttling of HNRNPC to the cytoplasm resulted in increased inclusion of YAP1 exon 4 (Figure 3A, right)—mimicking HNRNPC depletion conditions.^66^ Interestingly, the inclusion of YAP1 exon 4 increases in heart failure.^66,72^ This exon encodes one of two small modular protein domains containing two conserved tryptophan (W) residues known as WW domains, which promote YAP1 physical interactions with TEA domain (TEAD) transcription factors.^66,72,73^ YAP1 isoforms including exon 4 are more efficient at inducing transcription mediated by TEAD (Figure 3A, bottom right).^72^ Separate studies have linked YAP1 and TEAD to the transcription of genes involved in mesodermal specification, cardiac differentiation, cell proliferation, and focal adhesion assembly,^72,73^– and there is evidence that differential YAP1 isoform binding to TEAD is responsible for distinct transcriptome signatures.^72^ It can therefore be speculated that HNRNPC regulation of YAP1 alternative splicing influences transcriptional programs driven by TEAD.

In summary, ECM remodeling in response to cardiac insults controls HNRNPC regulation of alternative splicing and localization in cardiomyocytes. However, HNRNPC belongs to the HNRNP family of RBPs, and members of this family participate in multiple spheres of RNA biology, including transcription, mRNA stabilization, and translational regulation.^68^ Therefore, HNRNPC’s contribution to heart disease likely involves multifactorial mechanisms in addition to the regulation of mechanosensitive splicing. Future research should continue exploring the connections between HNRNPC and ECM remodeling because, in addition to cardiac pathologies, ECM remodeling is an important feature of tumor progression, immune escape, and vascular development and disease.^74–76^

### RBM4 regulates the Hippo pathway during tumor proliferation

The Hippo pathway can be mechanically activated by changes in cellular tension, cell attachment, fluid flow, and ECM stiffness.^50,77–85^ Within the Hippo pathway, the transcriptional co-activators YAP1 and WW domain containing transcription regulator 1 (WWTR1) (commonly known as TAZ) regulate the activation of TEAD proteins.^78,79^ When the Hippo axis is inactive, YAP1 and TAZ bind to TEADs in the nucleus and promote the expression of the TEAD transcriptional targets. When the Hippo axis is inactive, YAP1 and TAZ are phosphorylated, which leads to their cytoplasmic retention or degradation and, ultimately, the transcriptional silencing of TEAD targets.^78^ An alternatively spliced isoform of TEAD4, wherein skipping of exon 3 results in an alternative start site at exon 6 produces a truncated protein known as TEAD4-S.^69^ This alternative splicing event is regulated by the RBP RNA-binding motif 4 (RBM4).^69^ RBM4 is a tumor suppressor whose expression in multiple human cancers positively correlates with patient survival.^86^ RBM4 has been shown to regulate alternative splicing of multiple cancer-related genes, including TEAD4.^69,86^ RBM4 binds to a CGGCCGG motif within TEAD4 exon 3 and promotes its skipping, resulting in the expression of the short TEAD4-S protein isoform (Figure 3B, top).^69^ The TEAD4-S isoform lacks the N-terminal DNA-binding domain of TEAD but retains the YAP1 interaction domain; therefore, TEAD4-S is unable to regulate transcription but still interacts with YAP1 and TAZ.^69^ The interaction between TEAD4-S and YAP1 attenuates the activation of the Hippo-YAP1 pathway in mouse models of lung and colon cancer, downregulating YAP1 targets (Figure 3B, middle).^69^ The overexpression of TEAD4-S reduces cancer cell proliferation and migration, as well as epithelial-mesenchymal transition in lung cancer cells and in a xenograft tumor model.^69^ Thus, TEAD4-S expression slows cancer metastasis, improving survival (Figure 3B, bottom).^69^ In line with altering TEAD4 splicing, overexpression of RBM4 in multiple cancer cell lines and xenograft tumors results in reduced proliferation, migration, and apoptosis.^86,87^ Though it is important to note that high levels of RBM4 are surprisingly oncogenic in esophageal squamous cell carcinoma^88^ and acute myeloid leukemia,^89^ suggesting that the role of RBM4 in cancer is not ubiquitous across all cancer types.

In humans, TEAD4-S expression is reduced in cancerous versus healthy tissues.^69^ Individuals with lung and colon cancer and higher levels of TEAD4-S exhibit improved survival compared to patients with higher expression of the full-length TEAD4 transcripts.^69^ In this model, RBM4 binding to TEAD4 pre-mRNA promotes the production of the short TEAD4-S isoform which competes with the full-length TEAD4 variant for YAP1/TAZ binding, resulting in reduced transcription of genes encoding proteins involved in cell proliferation, migration, and apoptosis (Figure 3B).^69^ Cell stress induces RBM4 phosphorylation, which results in its translocation from the nucleus to cytoplasmatic stress granules.^90,91^ While in the nucleus, RBM4 regulates alternative splicing, whereas cytoplasmic RBM4 has different functions in RNA metabolism.^90,91^ Therefore, it can be speculated that mechanical changes in the tumor microenvironment, such as ECM stiffness, might cause cellular stress and drive RBM4 cytosolic localization.

### SR proteins in muscle and tumor microenvironment

Another class of RBPs implicated in mechanotransduction and alternative splicing regulation is the serine arginine-rich (SR) proteins. Phosphorylation of SR proteins facilitates their functions in splice site recognition.^92^ Our group has found that the phosphorylation of the serine-arginine-rich splicing factor 4 (SRSF4) responded to mechanical stretching in myotubes, and this was correlated with significant changes in alternative splicing of 41 SR protein targets.^93^ This indicates that SRSF4 acts as a regulator of mechanosensitive splicing networks in muscle cells.^93^

Similarly, SRSF5 was more phosphorylated when ECM stiffness increased in cultured endothelial cells and less phosphorylated when ECM stiffness was decreased in mouse mammary tumors *in vivo*.^94^ Interestingly, ECM stiffness correlated with the level of inclusion of an alternatively spliced region in the fibronectin pre-mRNA known as the extra domain B (ED-B).^94^ Inclusion of ED-B is prevalent in numerous types of aggressive solid tumors and is considered a biomarker of tumor angiogenesis.^95–97^ Endothelial cells plated on stiff substrates (10 kPa) exhibited higher levels of fibronectin variants containing ED-B versus total fibronectin than cells plated on compliant substrates (1 kPa). Moreover, mouse mammary tumors, where stiffness was reduced, expressed less ED-B containing fibronectin compared to control tumors.^94^ This study builds on the broader understanding that differences in ECM stiffness and composition influence splicing decisions in cultured cells and *in vivo*.^94,98–100^ A potential molecular player connecting SRSF5, fibronectin splicing, and mechanotransduction is the phosphoinositide 3-kinase/serine/threonine protein kinases (PI3K/Akt) pathway. The PI3K/Akt pathway regulates SR protein phosphorylation^101,102^ and the cellular response to ECM stiffness.^103,104^ When the PI3K/Akt axis was pharmacologically inhibited, SRSF5 phosphorylation was reduced and ED-B inclusion decreased, thus confirming a model wherein the PI3K/Akt signaling regulates SRSF5 phosphorylation, which thereafter controls fibronectin splicing in mammary tumors.^94^ A similar mechanism was also observed in human primary proximal tubule epithelial cells.^105^ In sum, it is becoming more evident that mechanical stimuli can somehow influence SR protein activity in multiple cell types, including myocytes and tumor cells.

### RBFOX1 regulates alternative splicing of proteins important for cardiac muscle contraction

The RNA-binding fox-1 or RBFOX family of proteins is widely known for its roles in regulating tissue-specific alternative splicing, particularly in the heart and skeletal muscle.^71,106^ RBFOX homolog 1 (RBFOX1) typically binds intronic (U)GGAUG motifs proximal to the regulated exons.^107,108^ When RBFOX1 binds upstream, the alternative exon is skipped, whereas when RBFOX1 binds downstream, the alternative exon is included.^71,109^ RBFOX1 is upregulated during cardiomyocyte differentiation and postnatal murine heart development (Figure 3C),^110^ and its expression is significantly suppressed in murine cardiac hypertrophy and heart failure (Figure 3C).^70^ Cardiac pressure overload induced distinct alternative splicing changes in cardiomyocyte-specific *Rbfox-1* knockout mice and transgenic mice overexpressing RBFOX1 than those observed in control mice.^70^ This suggests that RBFOX1 activity might regulate mechanosensitive alternative splicing programs in the heart. Interestingly, RBFOX1 overexpression prevented the development of pathological hypertrophy induced by pressure overload (Figure 3C, left), thus positing that RBFOX1 regulation of splicing is a driver of pathological cardiac hypertrophy.^70^ In zebrafish, *rbfox1* depletion caused a reduction in ventricular contractility and progressive heart failure, exemplifying the importance of RBFOX1 in cardiac contractility.^111^

More recently, RBFOX1 has been shown to regulate alternative splicing of several focal adhesion proteins, including paxillin and vinculin.^71^ Focal adhesions link the ECM to the cytoskeleton via integrin receptors.^112^ Bidirectional mechanical signals transmitted between cells and the ECM are processed at focal adhesions.^112,113^ Focal adhesions comprise (1) transmembrane integrin receptors that attach cells to other cells and/or to the ECM, (2) intracellular scaffolding proteins that interact with integrin receptors, and (3) actin-binding proteins that link focal adhesions to the actin cytoskeleton.^114^ In the focal adhesions, paxillin is an essential scaffolding protein that initiates the recruitment of other focal adhesion proteins and activates various downstream signaling pathways. Alternative splicing produces an extended paxillin variant that is predicted (via AlphaFold) to have additional intrinsically disordered regions, which contain binding motifs for several serine/threonine and tyrosine kinases and various Src family kinases.^71^ The vinculin encoding gene (*VCL*) undergoes tissue-specific alternative splicing, where inclusion of exon 19 produces the splice isoform metavinculin.^115–117^ Vinculin (–exon 19) is a mechanosensitive component of the focal adhesions that tethers actin filaments to the integrin receptor adaptor talin to regulate force-sensitive cellular events.^118,119^ Metavinculin (+exon 19) is an extended splice isoform of vinculin mostly expressed in myocytes that also function in focal adhesions, but with different properties in terms of actin filament binding (see section entitled “Alternative splicing of fibronectin, a mechanosensitive ECM protein”). RBFOX1 depletion in H9c2 cardiomyocytes resulted in decreased expression of metavinculin and decreased inclusion of paxillin exon 7 when compared to cells overexpressing RBFOX1 (Figure 3D).^71^ Functionally, RBFOX1 depletion in cardiomyocytes resulted in reduced differentiation indicated by downregulation of myogenic markers and fewer multinucleated cells compared to controls (Figure 3E).^71^ Conversely, cells overexpressing RBFOX1 exhibited substantially altered focal adhesion structures and cytoskeleton organization, resulting in elongated cells (Figure 3E).^71^ In summary, RBFOX1 is an important regulator of focal adhesion assembly during cardiomyocyte differentiation, and its downregulation in cardiac hypertrophy and consequential disruption of focal adhesion assembly may contribute to disease progression.

### Mechanical cues regulate PTBP1 subcellular localization

A recently published screen to identify proteins involved in mechanotransduction via proteomics revealed that the subcellular localization of the polypyrimidine tract binding protein 1 (PTBP1) is mechanosensitive, responding to differences in cell density, cell area, and ECM stiffness.^120^ Because PTBP1 is a well-known splicing factor,^121^ researchers further assessed global changes in alternative splicing after PTBP1 depletion. One interesting splicing change that they identified occurred in exon 9 of the NUMB endocytic adaptor protein (Numb) pre-mRNA, which was more skipped in PTBP1-depleted cells than in controls.^120^ Numb exon 9 was also mostly skipped in MCF10A cells cultured on low stiffness polyacrylamide gels (0.2 kPa) but mostly included in cells cultured on high stiffness gels (25 kPa). Changes in Numb exon 9 splicing correlated with PTBP1 nuclear localization and relative PTBP1 protein abundance in cells plated on a 25 kPa polyacrylamide gels versus 0.2 kPa gels,^120^ leading authors to propose that PTBP1 may regulate Numb alternative splicing in a mechanosensitive manner. This claim was further strengthened by the observation that PTBP1-depleted cells recovered their proliferation capacity upon re-expression of the long NUMB isoform (+exon 9) but not the short NUMB isoform (–exon 9).^120^ Furthermore, cells treated with antisense oligonucleotides targeting Numb exon 9 exhibited reduced cell spreading, while overexpression of the long NUMB isoform resulted in increased cell spreading on a soft matrix.^120^ Lastly, Numb exon 9 was required for integrin β1 recycling, which is a critical mechanotransduction mechanism that regulates the number and size of focal adhesions in a cell.^120,122,123^ Overall, this PTBP1 study is recent proof that the mechanical properties of the extracellular environment can inform RBP activity.

## NUMEROUS MECHANOSENSITIVE PROTEINS ARE ALTERNATIVELY SPLICED

Over 90% of multi-exonic human genes undergo alternative splicing; therefore, it is no surprise that several genes encoding proteins involved in mechanotransduction pathways are alternatively spliced. This section will focus on alternatively spliced mechanosensors and mechanotransducers that modulate the cellular response to mechanical stimuli. Table S1 summarizes what is currently known about the mechanosensitive proteins discussed in this section: splice variants and/or alternatively spliced regions, their regulators (if known), affected pathways, and associated phenotypes and/or diseases. It is important to note that the splicing field has made enormous progress on molecularly identifying alternatively spliced transcripts, but we still lack that same level of understanding about two downstream aspects: (1) whether different transcripts are translated into distinct protein isoforms and (2) the common and unique functions of these different protein isoforms, when produced. Addressing these questions is not trivial because it first requires us to establish what to assay in terms of protein function. This usually starts with the known roles of the protein under investigation, which usually come from preexisting research on the most common or only studied splice variant. Second, not finding differences between splice variants in those assessed specific functions does not rule out that the other splice isoforms might have functions that were not yet discovered. Finally, all these attempts often start in cell culture systems; the cases where this goal has been accomplished within *in vivo* conditions are a minority, though extremely significant. This is one of the main active challenges in the splicing field. Wherever possible, we will highlight studies that have used multiple experimental models.

### Alternative splicing of fibronectin, a mechanosensitive ECM protein

ECM composition influences the stiffness of the extracellular environment and the activation of mechanotransduction pathways.^9^ One of the major ECM components is fibronectin, a large glycoprotein that interacts with integrin receptors. It is secreted by multiple cell types,^9,124,125^ mediates wound healing and tissue damage repair, and plays active roles in embryonic development.^125^ The interactions between integrin receptors and fibronectin are particularly important for mechanotransduction, as this binding enables cells to sense and adapt to changes in ECM stiffness.^126^

Fibronectin was one of the first genes to be studied in terms of its ability to be alternatively spliced.^29,127–130^ However, only more recently, discoveries have illuminated the role of fibronectin splice isoforms in arterial wall composition and hemodynamics,^131,132^ cardiovascular development,^133^ tumor microenvironment,^134,135^ renal fibrosis,^136^ and innate immunity,^137^ among others. All these physiological states rely heavily on mechanotransduction for proper tissue homeostasis.^52,131,132,138^ There are at least 20 fibronectin splice variants in humans generated by the inclusion or exclusion of three regions: the ED-A, the ED-B, and the type III connecting sequence (or variable region).^127,139–141^ Here, we will focus on the ED-A and ED-B because alternative splicing of these regions is heavily associated with human diseases.^142^ ED-A and ED-B are encoded by cassette exons that can be either skipped or included. Plasma fibronectin is a soluble form found in blood plasma, predominantly synthesized by the hepatocytes.^143^ Basally, ED-A and ED-B are skipped in plasma fibronectin transcripts.^143^ In contrast, cellular fibronectin is an insoluble ECM component that is produced by a wide range of cell types.^143^ Cellular fibronectin displays much more isoform diversity than plasma fibronectin.^29,144,145^ The alternatively spliced exons encoding ED-A and ED-B are gradually included through embryogenesis but are skipped in most adult tissues.^140,144–147^ The inclusion of these cassette exons increases in circulating plasma and the vascular ECM after injury^148–150^ and during wound healing.^145,151–153^ While it is frequent for ED-A and ED-B to display similar trends in alternative splicing, there is also evidence of cells that produce transcripts containing ED-A or ED-B alone,^147,154–156^ suggesting that ED-A and ED-B splicing is independently regulated.

Decades of research on fibronectin splicing have identified the *cis*-elements and *trans*-acting factors regulating ED-A and ED-B splicing. The intron downstream of ED-B exon contains numerous UGCAUG hexanucleotide motifs that contribute to ED-B inclusion.^157,158^ This UGCAUG sequence is the consensus binding motif for RBFOX2.^60,108,159^ When RBFOX2 binds these motifs downstream of the ED-B exon, it promotes its inclusion.^160–162^ RBFOX2 can also promote ED-A inclusion, but its depletion results in a less dramatic shift compared to the effect on ED-B splicing, suggesting that other RBPs might contribute to the regulation of ED-A splicing.^160,161^

SRSF1 is indeed one regulator of ED-A splicing. Differential expression of SRSF1 in endometrium and dermis fibroblasts in the uterus contributes to the regulation of trophoblast invasion.^163^ In endometrial fibroblasts, SRSF1 is highly expressed, resulting in increased ED-A inclusion.^163^ In contrast, dermis fibroblasts express low levels of SRSF1 and, therefore, ED-A is predominantly skipped.^163^ This difference in fibronectin splicing is correlated with the ratio of ECM fibronectin versus soluble fibronectin: high ECM fibronectin in the endometrium and high soluble fibronectin in the dermis.^163^ As a consequence of ECM fibronectin levels, the endometrium is an environment prone to trophoblast invasion, whereas the dermis is resistant to cell invasion.^163^

The RBP Quaking (QKI) can also repress ED-A inclusion by binding to upstream intronic sequences.^137^ In QKI-depleted cells, fibronectin protein containing ED-A increases,^137^ which contributes to the upregulation of the interferon response and the reduced production of viruses upon infection.^137^

Regulation of fibronectin splicing is mechanosensitive. Endothelial cells lining the vasculature are highly sensitive to shear stress from the blood flow. The level of exposure to endothelium shear stress is one of the best predictors of atherosclerotic lesion size and vulnerability, as well as aneurysm growth and rupture.^164–167^ Generally, aneurysm growth and atherosclerotic plaques develop in vessels exposed to relatively low flow.^166,167^ In mice, endothelial cells exposed to low flow exhibited an increase in ED-A and ED-B inclusion without a change in overall fibronectin expression.^131^ This response observed under low flow wherein hematopoietic cells are recruited to the arterial endothelium resulted in global changes in alternative splicing which are partially regulated by RBFOX2.^161^ Indeed, mice with endothelial cell-specific deletion of RBFOX2 subjected to partial carotid ligation (to induce low flow) exhibited 50% ED-A inclusion compared to 60% in wild-type littermates (arteries under high flow conditions had 20% ED-A inclusion).^161^ In contrast, ED-B was completely skipped in RBFOX2-depleted endothelial cells in all the tested flow conditions.^161^ These results point to RBFOX2 being a major regulator of ED-B splicing, whereas ED-A inclusion is mediated by other RBPs.

Moreover, when mice unable to express ED-A and ED-B were exposed to low flow, more severe and frequent localized hemorrhages were formed in the blood vessels than those in wild-type littermates.^131^ These findings suggest that ED-A and ED-B inclusion in blood vessel endothelial cells is protective against intimal hemorrhage. Fibulin-1 is an ECM and blood plasma protein that deposits around atherosclerotic lesions in arterial walls,^168^ and this recruitment is dependent on the expression of fibronectin splice variants containing the ED-A and ED-B domains.^132^ Considering that reduced fibulin-1 in the circulating blood serum correlates with the early onset of atherosclerosis,^169^ the above finding suggests that ED-A and ED-B inclusion exerts a protective effect against the development of vascular diseases.^132^ These studies on fibronectin splicing in blood vessels are an excellent example of how mechanical forces (laminar flow) impact alternative splicing of a mechanosensitive protein (fibronectin) and also show the reverse, where fibronectin alternative splicing impacts how cells respond to changes in flow. Finally, we also described that fibronectin splicing is also mechanosensitive in tumor endothelial cells (see previous section entitled “RBFOX1 regulates alternative splicing of proteins important for cardiac muscle contraction”), though it is interesting that, in that case, ED-B is skipped in the tumor microenvironment that has low stiffness.^94^ Given that these studies (blood vessel endothelial cells and tumor endothelial cells) investigated different RBPs, RBFOX2 and SRSF5, respectively, it is possible that the expression of specific RBPs dictates alternative splicing outcomes in endothelial cells present in distinct tissue environments.

### Alternative splicing of Piezo proteins regulates their functions as mechanosensors

Piezo proteins are components of mechanically activated ion channels that form pores in the plasma membrane to enable cation transport. Piezo1 and Piezo2 are the two Piezo proteins conserved in mammals.^34^ Both Piezo1 and Piezo2 form homotrimeric complexes known as Piezo1 or Piezo2 channels.^170^ Piezo channels play essential roles in physiology: (1) urothelium sensation of bladder extension,^171^ (2) sensation of compressive force in articular cartilage chondrocytes,^172^ (3) regulation of red blood cell volume,^173,174^ (4) sensation of sheer stress in the vasculature,^175^ (5) touch sensation by Merkell cells,^176–178^ and (6) fluid filtration by kidney podocytes.^179^

Alternative splicing regulation of the mammalian Piezo proteins alters their sensitivity to mechanical forces. Piezo1.1 is an evolutionarily conserved splice isoform of Piezo1, which is produced by skipping exon 30 from the Piezo1 transcript.^180^ Piezo1.1 is broadly expressed across human and mouse cell lines and tissues, though the ratio of Piezo1.1 to Piezo1 expression differs between cell and tissue types.^180^ The structural difference between Piezo1 and Piezo 1.1 isoforms is due to Piezo1.1 lacking a 24-amino-acid-long sequence within the intracellular “lateral plug domain” of Piezo1.^180^ This lateral plug domain functions as a barrier regulating cation transport through the ion-conduction pore of Piezo1 channels.^180^ Because of this structural difference, the Piezo1.1 isoform (+exon 30) is more sensitive to stretch-activated currents than Piezo1 isoform (–exon 30) and has reduced calcium ion permeability in HEK cells; thus, alternative splicing directly impacts Piezo1 function during mechanotransduction.^180^ Therefore, Piezo1 and Piezo1.1 are proposed to form homomeric and heteromeric channels with different levels of conduction.^180^ What remains unknown is how splicing regulation of Piezo1 influences mechanosensation and cation permeability in different cell types.

Piezo2 is also alternatively spliced at its exon 33, which encodes a region homologous to the Piezo1 lateral plug domain.^180,181^ Cell-type-specific splicing of Piezo2 is better understood than that of Piezo1 splicing. In mice, the sensory neurons in the trigeminal ganglion express at least 17 Piezo2 isoforms containing different combinations of five alternatively spliced exons (exons 33, 18, 19, 35, and 40).^181^ Conversely, “non-sensory tissues” such as the lung and bladder only express one prevalent Piezo2 variant.^181^ Even more complexly, Piezo2 alternative splicing patterns are not the same across all sensory neurons. Sensory neurons involved in nociception (those expressing the transient receptor potential vanilloid 1 ion channel, TRPV1) predominantly express Piezo2 transcripts lacking exon 35.^181^ In contrast, neurons involved in discriminative touch and proprioception (those not expressing TRPV1) mostly express Piezo2 transcripts including exon 35.^181^ Additionally, non-neuronal Merkel skin cells involved in touch sensation also express Piezo2 isoforms containing exons 33 and 35.^181^ This evidence suggests that the expression of additional Piezo2 splice variants is important for the biophysical properties of Piezo channels and particularly for properties controlling neuronal excitability and touch sensation.^181^

Szczot and colleagues sought to characterize the properties of neuronal versus non-neuronal Piezo2 variants.^181^ To do this, researchers focused on the predominant non-neuronal Piezo2 variant that includes the alternative exons 18, 19, 35, and 40 but skips exon 33 (referred to as Piezo2-V2) and the predominant neuronal Piezo2 variant that skips exons 18, 19, 35, and 40 but includes exon 33 (referred to as Piezo2-V14).^181^ HEK293 cells transfected with the Piezo2-V2 variant exhibited increased calcium ion permeability and increased rates of Piezo2 inactivation when compared to cells transfected with the Piezo2-V14 variant.^181^ This is important because the rate of Piezo pore closure during channel inactivation contributes to the frequency filtering of repetitive stimuli such as mechanical vibration.^182^ Overall, the findings discussed in this section have demonstrated that alternative splicing is a mechanism to tune the mechanosensing and the biophysical properties of Piezo1 and 2. At the time of this review, the articles discussed here are the only two that explored Piezo alternative splicing. An intuitive next step in the field would be to assess the functional implications of Piezo isoform expression in more physiologically relevant cell types and tissues using *in vivo* models. Moreover, due to this same limited scope of publications, the field has not yet identified the regulators of Piezo splicing.

### TREK-1 splice isoforms exhibit different localization at the plasma membrane and conductance capacity

Similar to Piezo channels, other transmembrane proteins are mechanically activated. The potassium two-pore domain channel subfamily K member 2 (*KCNK2*) gene encodes the mechano-gated potassium channel called TREK-1. TREK-1 regulates cell membrane electrophysiology during uterine quiescence,^183,184^ in the heart^185^ and in the peripheral^186^ and central nervous systems.^187^ Alternative splicing of the *KCNK2* gene produces different mRNA transcripts, some of which result in TREK-1 protein isoforms. TREK-1a, TREK-1b, and TREK-1c are the first three identified splice variants of TREK-1, and all three are expressed in the human brain.^188–192^ TREK-1a and TREK-1b are also present in the heart,^191^ and so far, no biophysical differences have been detected.^191^ There is an additional TREK-1d variant that is restricted to rats.^192^ All these variants have N-terminal regions generated by alternative splicing of exons 1 and 2.^188–192^ There is an additional TREK-1e variant that was identified more recently that skips exon 5 and has a premature stop codon, resulting in a non-functional TREK-1 protein.^192^ TREK-1e is expressed in rat and human brains, kidneys, and adrenal glands.^192^ Co-expression of TREK-1e with TREK-1a resulted in reduced expression of TREK-1a and reduced TREK-1a currents, suggesting that the function of this splice variant is to attune and regulate production of functional TREK-1.^192^

In the human myometrium, TREK-1a, TREK-1b, and TREK-1c are expressed during non-pregnancy and pregnancy conditions.^188^ Additional novel variants of TREK-1 were identified in myometrium from women who experienced spontaneous preterm labor (or preterm delivery of an underdeveloped fetus at 26–34 weeks in pregnancy).^188^ These transcript variants named SV-1 (+exons 1, 4, 5, 6, 7, and 8), SV-2 (+exons 1, 5, 6, 7, and 8), SV-3 (+exons 1, 5, 7, and 8), SV-4 (+exons 1, 7, and 8), and SV-5 (+exons 1 and 8) produce truncated TREK-1 proteins.^184,188^ Expression of these short variants in the myometrium correlated with risk for preterm labor: mothers experiencing normally timed labors expressed less of the short variants compared to mothers undergoing preterm labor.^184,188^ These findings are clinically important because they seem to indicate that TREK-1 variants SV-1, SV-2, SV-3, SV-4, and SV-5 play a pathological role in preterm labor.^184,188^

Studies in cell culture revealed differences in the functions and localization of TREK-1 splice variants. Specifically, HEK293T cells expressing individual TREK-1 short variants exhibited decreased KCNK2 mRNA expression, reduced localization of TREK-1 at the plasma membrane, and reduced TREK-1 function compared to cells expressing the full-length TREK-1 isoform.^184^ Truncated TREK-1 variants tend to have a similar role in sequestering TREK-1 protein in the cytoplasm. Another functional study performed by transfecting a transformed human kidney cell line with TREK-1 variants investigated a novel and not previously described brain-specific truncated TREK-1 variant lacking KCNK2 exon 4 (TREK1ΔE4). This variant was not able to conduct currents, and its co-expression with the equivalent wild-type TREK-1 form, including exon 4, resulted in reduced cell conductance.^193^ This dominant negative effect of TREK1ΔE4 was due to either impaired trafficking of functional TREK-1 to the plasma membrane or inhibitory effects caused by the binding of the truncated TREK-1 variant to the functional TREK-1.^193^

Although these studies have provided great evidence for the role of splicing regulation in TREK-1 functions, they were all performed in cells. Further research is needed to fully understand how TREK-1 splicing regulates the *in vivo* electrophysiology of tissues where TREK-1 is expressed, such as the brain, myometrium, heart, lung, kidney, adrenal gland, and skeletal muscle.^185,189,192^ Studies addressing how myometrial TREK-1 splicing is dysregulated in preterm myometrium could be instrumental in understanding basic mechanisms and potentially developing therapeutics to prevent preterm deliveries. Additionally, TREK-1 activity is important for cardiomyocyte response to pressure overload,^194^ pain perception,^186^ brain ischemia,^195^ and morphine analgesia.^196^ Despite TREK-1 undergoing alternative splicing in both neurons and the heart, much remains unknown about the roles of TREK-1 isoforms in modulating TREK-1 activity in cardiovascular disease and in the central and peripheral nervous systems *in vivo*.

### Vinculin and its splice variant metavinculin have unique mechanical properties

Vinculin is an action-binding protein that acts as a molecular force transducer by connecting actin filaments to the integrin receptor activator talin.^118,119,197,198^ The interaction between vinculin and talin is mechanically induced^118,199–201^ and vinculin binding to filamentous actin is necessary for the propagation of mechanical signals by focal adhesions.^200,202^

In metavinculin, the 68-amino-acid insert encoded by exon 19 results in an extended coil domain and modified first alpha helix in the actin-binding domain situated in the C-terminus.^203,204^ Crystal structures and biochemical analysis revealed that this alternatively spliced domain gives metavinculin distinct actin binding and oligomerization capabilities compared to vinculin.^203,205–208^ These structural discoveries open the question: what is the physiological significance of the metavinculin splice isoform?

To first address this question, several groups analyzed meta-vinculin expression in different cell types. While vinculin is ubiquitously expressed, metavinculin expression is predominantly limited to myocytes.^115,117,209^ Metavinculin and vinculin are co-expressed in smooth and striated muscle cells (though at different molar ratios) and colocalize to dense plaques, costameres, and intercalated discs—all of which are myocyte-specific adhesion sites.^115,116,209,210^ Colocalization of metavinculin and vinculin at muscular junctions suggests that they cooperatively modulate force transmission in muscle.^209,211,212^ The speculated purpose of the cooperation between vinculin and metavinculin is that constant muscle contraction makes myocytes more susceptible to damage from mechanical forces, requiring additional structural support to maintain their cellular integrity during contraction. Moreover, mutations in exon 19 are present in humans with dilated and hypertrophic cardiomyopathy,^211–213^ individuals with these mutations exhibit intercalated disc defects, and the mutant proteins bind actin filaments differently than wild-type vinculin or metavinculin.^211,212^ Therefore, metavinculin expression in cardiomyocytes seems to be critical for cardiac health.

Though vinculin and metavinculin are co-expressed,^115,117,214^ there is evidence for isoform-specific functions. Only vinculin can form actin filament bundles,^215^ probably because the differences in the C-terminal domain result in differences in actin filament binding between metavinculin and vinculin.^205–208^ Moreover, the alternative C-terminus tail domain in metavinculin leads to stronger binding affinity with talin compared to vinculin.^216^

The specific functions of metavinculin are the focus of recent publications and ongoing investigations. To distinguish between isoform-specific functions at focal adhesions, Lee and colleagues stably expressed vinculin or metavinculin in *Vcl*-null mouse embryonic fibroblasts.^215^ Metavinculin expression rescued cell area reductions previously observed in *Vcl*-null fibroblasts,^200^ and metavinculin-expressing fibroblasts formed larger but fewer focal adhesions than vinculin-expressing fibroblasts.^215^ Moreover, fibroblasts expressing metavinculin exhibited reduced cellular migration and reduced cell stiffening response when compared to fibroblasts stably expressing vinculin.^215^ In addition to RBFOX1^71^ (see section entitled “RBPs can regulate RNA processing during mechanotransduction”), alternative splicing of exon 19 can also be regulated by RBM10.^217^ In thyroid cancer cells, metavinculin is aberrantly expressed due to RBM10 depletion.^217^ In addition to aberrant metavinculin expression, RBM10 depletion caused dysregulated splicing of other cytoskeletal and ECM proteins in thyroid cancer cells, which resulted in increased cell velocity and metastatic properties both *in vitro* and in mouse models.^217^

A very interesting study measured the mechanical properties of metavinculin and vinculin in cells using piconewton-sensitive tension sensors.^216^ Intriguingly, despite having a stronger binding affinity to talin, metavinculin was less efficient at transducing mechanical forces than vinculin in mouse fibroblasts, HEK293 cells, and HL-1 cardiomyocytes.^216^ This result points to metavinculin potentially playing a role in buffering cells from sudden changes in extracellular stiffness.^216^ Therefore, it is expected that changes in metavinculin expression in cardiomyocytes might compromise the structural integrity of the heart and make subjects more susceptible to cardiac diseases. This prediction is consistent with the previously mentioned findings in dilated and hypertrophic cardiomyopathy patients with loss-of-function mutations in metavinculin.^211,212^ Perplexingly, transverse aortic constriction in mice resulted in similar levels of cardiac hypertrophy in metavinculin knockout mice and wild-type littermates.^216^ When compared to wild-type mice, metavinculin knockout animals appeared to have unchanged intercalated disks based on β-catenin and metavinculin/vinculin staining.^216^ These findings refute the hypothesis that metavinculin expression is essential for cardiac muscle structure and function in health and disease, at least in mouse models. Since metavinculin and vinculin are co-expressed in myocytes, it is possible that their cooperative functions are important in muscle cell mechanotransduction. Overall, the studies described in this section revealed that metavinculin and vinculin have distinct mechanical properties likely due to differences in the structure of their C-terminus tail domain.

### Alternative splicing regulates multiple transmembrane components of the hair cell mechanotransduction complex

The inner ear, or cochlea, contains auditory hair cells whose function is to convert sound (a mechanical stimulus) into electrical signals that are propagated by neurons.^37^ Stereocilia are specialized microvilli structures that serve as the mechanosensing organelles of hair cells.^37^ Hair bundles are arrays of stereocilia organized in a gradient of increasing lengths and widths to enhance their ability to intercept sounds.^37,218^ It is well accepted that hair cell mechanotransduction is initiated by deflections in hair bundles, which thereafter cause bending and shearing of adjacent stereocilia.^218,219^ Mutations in genes encoding proteins that regulate hair bundle development and maintenance cause deafness, demonstrating the importance of mechanotransduction in hair cells.^220^

The molecular mechanisms responsible for hair cell mechanotransduction are still under investigation; therefore, it is only reasonable that our understanding of the role of splicing in this context is still developing. The hair cell mechanotransduction complex, which includes a mechanosensitive ion channel that is sensitive to hair bundle deflection and other molecular players, is positioned at the apical tip of stereocilia and converts sound waves into electrical signals.^37,218,221,222^ A plethora of molecules are required for mechanotransduction in cochlear hair cells, though their specific functions are still under investigation.^37^ Four components of the hair cell mechanotransduction complex are transmembrane proteins that undergo alternative splicing are as follows: (1) transmembrane channel-like protein 1 (TMC1), (2) protocadherin 15 (PCDH15), (3) lipoma HMGIC fusion partner-like 5 (LHFPL5), and (4) transmembrane inner ear protein (TMIE).^223–225^

TMC1 forms the core of mechanoelectrical transduction channels at the tips of stereocilia in inner ear hair cells, making its expression essential for hearing in mice and humans.^221,226^ TMC1 is a transmembrane protein with 10 transmembrane domains that dimerizes to form an ion channel-like structure around hair cell sensory architecture.^227^ *TMC1* mutations cause hereditary deafness in humans and mice.^226,228^ In mouse cochleae, Tmc1 mRNA expression is undetectable at postnatal day 2 and dramatically increases 20-fold at postnatal day 5 and 200-fold by postnatal day 22.^221^ Moreover, when Kawashima and colleagues isolated cochlear hair cells from neonatal mice (postnatal days 5–7), they observed an absence of detectable mechanotransduction currents in hair cells lacking TMC1 despite their normal morphology on scanning electron micrograph.^221^ These results mirror young mice (postnatal days 12–20) with homozygous recessive mutations in the “deafness gene,” which Kawashima et al. speculated was indeed *Tmc1* because mice with “deafness gene” mutations were completely deaf with no cochlear potential despite normal hair cell architecture.^229,230^ These studies suggest that TMC1 involvement in mechanotransduction is required for hearing in adult mice. Three alternative splicing events have been identified in TMC1 pre-mRNA.^221,224^ First, TMC1 exon 9 can be included or skipped. Second, TMC1 exon 14 contains an alternative 3^′^ splice site.^224^ Third, the TMC1A and TMC1B isoforms are generated from the presence of an alternative start codon wherein the transcript either starts at exon 1 and skips exon 2 in TMC1A or skips exon 1 and starts at exon 2 in TMC1B.^221,224^ In HEK293T cells transfected with the cDNA containing the sequence for TMC1A or TMC1B, only TMC1A transcripts were translated into TMC1 protein, while the TMC1B construct was unable to produce the TMC1 protein.^231^

Alternative splicing events were also identified for LHFPL5 and TMIE pre-mRNAs via *in-silico* analysis of RNA-sequencing data from isolated mouse cochlear inner and outer ear hair cells.^224,232^ LHFPL5 tethers the hair cell mechanotransduction channel proteins to tip links via its N-terminus at the tips of stereocilia, and this LHFPL5 function is necessary for normal mechanotransduction.^233,234^ Alternative splicing occurs at Lhfpl5 exon 2 where there is an alternative 3^′^ splice site.^224,235^ TMIE, TMC1, and TMC2 are subunits of cochlear hair cell mechanotransduction channels, where TMIE regulates the gating properties of the channel and is required for the proper functioning of the other two channel subunits.^236^ Tmie pre-mRNA contains an alternative 3^′^ splice site within exon 4.^224^ These TMC1, LHFPL5, and TMIE alternative splicing events were present in both the outer and inner ear hair cells, though the levels of inclusion of the alternative regions were ~10% or less in the original RNA-sequencing dataset.^224^ Analysis of Tmc1 and Lhfpl5 splicing in the cerebrum, cerebellum, cochlea, colon, eye, and testis— all of which express both Tmc1 and Lhfpl5— revealed that Tmc1 exon 9 skipping only occurred in the cochlea and testis, while the use of the alternative 3^′^ splice site in Lhfpl5 exon 2 occurred in the cochlea, testis, and colon^150^ demonstrating that these splicing events are tissue specific.^224^ It has yet to be assessed whether these newly identified transcripts are translated into alternative protein isoforms, though Zhou et al. have speculated on the potential outcomes based on amino acid sequence.^224^ Overall, these findings highlight the previously unappreciated complexity of the hair cell mechanotransduction machinery, and future work should further expand upon the functions of TMC1 and LHFPL5 isoforms in normal hearing and hearing loss.

PCDH15 and cadherin 23 form tip links, which are structures that act as extracellular linkages between adjacent stereocilia.^220^ Tip links are responsible for regulating tension between stereocilia so that the highly sensitive hair cell mechanotransduction complex is not always activated.^237,238^ There are four major classes of PCDH15 splice isoforms that are distinguished by their unique cytoplasmic domains (PCDH15-CD1, PCDH15-CD2, PCDH15-CD3, and PCDH15-SId).^223,239^ PCDH15-CD1, PCDH15-CD2, and PCDH15-CD3 isoforms are generated by the insertion of the following alternatively spliced exons: (1) exon 35 in PCDH15-CD1, (2) exon 38 in PCDH15-CD2, and (3) exon 39 in PCDH15-CD3.^223,239^ CDH15-SId transcripts are generated by the skipping of exons 27 through 39, which encode the transmembrane and cytoplasmic domains.^223^ The cytoplasmic domains of PCDH15-CD1, PCDH15-CD2, and PCDH15-CD3 isoforms have unique amino acid sequences.^223,239^ In addition to the cytoplasmic domain differences, these three isoforms also have distinct C-terminal amino acid sequences: PCDH15-CD1 has a serine-threonine-serine-leucine (STSL) sequence, PCHD15-CD2 has an asparagine-thre-onine-alanine-leucine sequence, and PCHD15-CD3 has a methi-onine-threonine-lysine-leucine sequence.^223^ Due to the lack of transmembrane and cytoplasmic domains present in the other PCDH15 isoforms, PCDH15-Sld variant is preferentially secreted by transformed natural killer cells derived from the lymphatic system.^223,240^ The PCDH15-SId isoform is also expressed in the mouse inner ear and is speculated to be secreted by cells comprising the inner ear.^223^ However, it remains unknown whether PCDH15-SId is involved in hair cell mechanotransduction. PCDH15-CD1 and PCDH15-CD3 localization at the stereocilia (where tip links form) suggests that they can form tip links in mature hair cells.^223^ Conversely, PCDH15-CD2 is distributed across the length of stereocilia during early hair bundle development but then becomes concentrated at the tip of hair bundles before its expression in stereocilia is undetectable in mature hair cells.^223^

Further, isoform-specific knockout of PCDH15-CD1, PCDH15-CD2, or PCDH15-CD3 in mice revealed that PCDH15 isoform expression directly impacts tip link formation and hearing.^239^ Depletion of PCDH15-CD1 or PCDH15-CD3 did not impact the development of functional tip links, and, therefore, mice lacking PCDH15-CD1 or PCDH15-CD3 had normal hearing.^239^ In contrast, adult mice lacking the PCDH15-CD2 isoform did not develop tip links between cilia and were subsequently deaf.^239^ A separate study further showed that PCDH15-CD2 isoform expression is the most important for mature mouse cochlear hair cell function, whereas immature hair cells can form tip links with any of the three PCDH15 isoforms.^225^ These results agree with findings in humans, where mutations impacting PCDH15-CD2 expression were found in individuals with hearing loss.^225^ Finally, recent work from Wang and colleagues has shown that RBM24 regulates PCDH15 splicing in the mouse inner ear, and that hair cell-specific knockout of RBM24 disrupts hair bundle development.^241^ In summary, PCDH15 is a critical mechanosensing component in hair cells that undergoes developmentally regulated alternative splicing via the action of RBM24 to produce a functional protein isoform required for the development of functional hair cells and proper hearing.

## MECHANOSENSITIVE RNA PROCESSING PROGRAMS

### Mechanosensitive splicing networks in skeletal muscle and kidney

**Figure 4.**
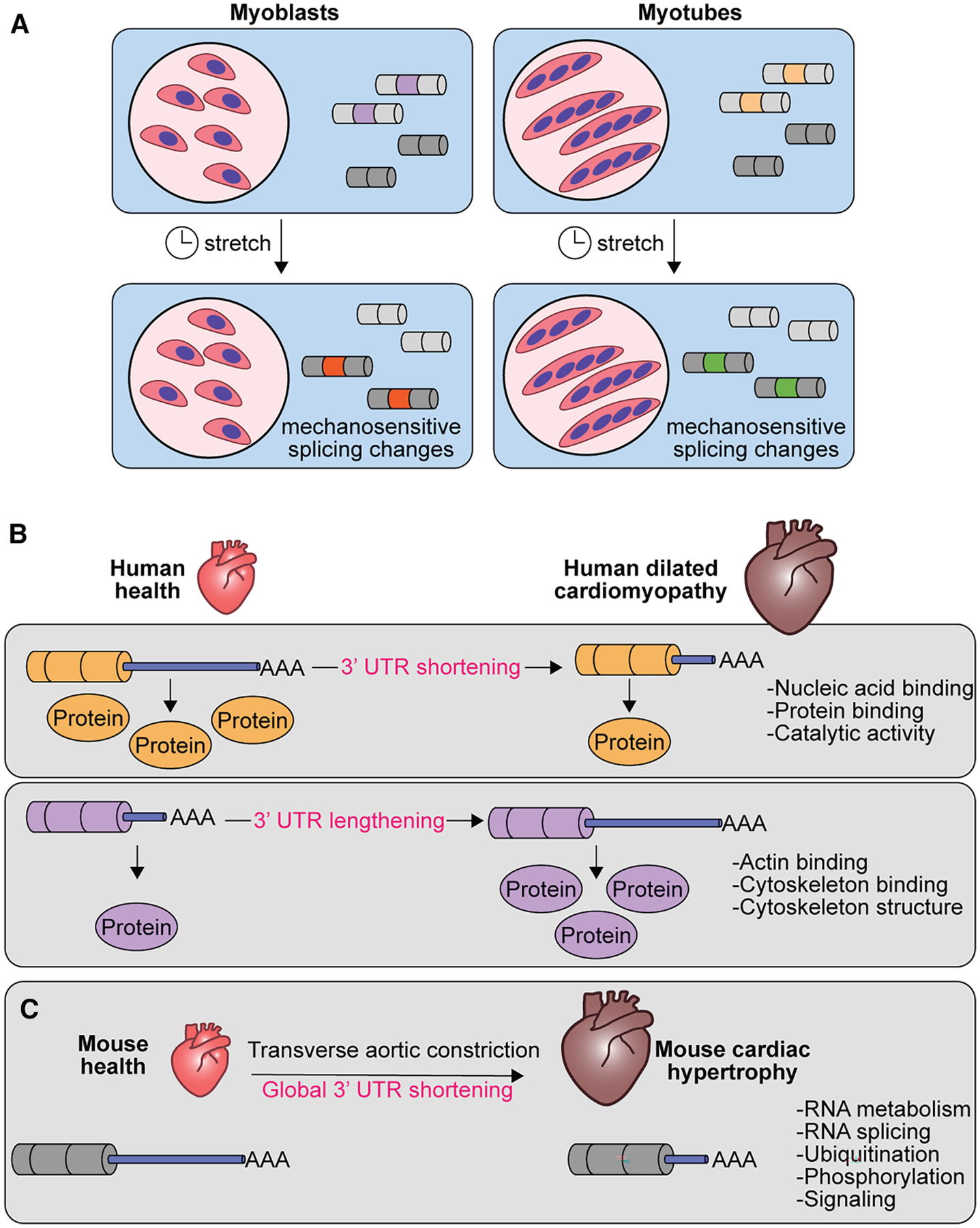
Alternative splicing and alternative polyadenylation networks can be mechanosensitive (A) Cultured C2C12 myoblasts and myotubes exhibited distinct alternative splicing changes in response to increasing time of equiaxial stretching. This schematic is based on findings published in Hinkle et al.^93^ (B) Healthy human hearts and hearts with dilated cardiomyopathy exhibit differences in 3^′^ UTR lengths for specific genes. Transcripts encoding proteins that function in nucleic acid binding, protein binding, and catalytic activity had shortened 3^′^ UTRs in dilated cardiomyopathy compared to healthy hearts (yellow). In this group, shortened 3^′^ UTRs corresponded with reduced translation into protein. Transcripts encoding proteins involved in actin binding, cytoskeleton binding, and cytoskeleton structure had longer 3^′^ UTRs in dilated cardiomyopathy compared to a healthy heart (purple). For candidates assessed from this group, longer 3^′^ UTRs corresponded with higher levels of translated proteins. This schematic is based on findings published in Creemers et al.^248^ (C) Mouse hearts that underwent transverse aortic constriction exhibited cardiac hypertrophy and differences in alternative polyadenylation compared to healthy hearts. The overall cardiac hypertrophy resulted in global 3^′^ UTR shortening, with the greatest changes occurring in transcripts encoding proteins implicated in RNA metabolism, RNA splicing, ubiquitination, phosphorylation, and signaling. This schematic is based on Soetanto et al.^249^

Alternative splicing programs regulate development, tissue identity acquisition, and disease progression.^29^ The brain, heart, and skeletal muscle are the tissues with the highest levels of evolutionarily conserved and tissue-specific alternative splicing.^242^ In skeletal muscle, disruption of global splicing programs or single alternative splicing events impairs muscle function.^23,243–247^ Because skeletal muscle is a highly mechanosensitive tissue, this raises the question of whether mechanical forces can induce splicing transitions in muscle cells. Recently, our group has reported that mechanical stretching resulted in global changes in transcription and alternative splicing in C2C12 mouse skeletal muscle cells.^93^ We have observed that distinct changes in splicing corresponded to increased stretching time in undifferentiated and differentiated C2C212 cells (myoblasts and myotubes, respectively) (Figure 4A).^93^ The observation that myoblasts and myotubes showed distinct responses to mechanical stretch at the level of splicing and transcription suggests that muscle cell differentiation state influences mechanotransduction responses. Given the importance of alternative splicing and mechanotransduction in skeletal muscle physiology, future studies should assess the functional consequences of the activation of mechanosensitive splicing networks in healthy muscles and in muscular diseases.

More recently, analysis of transcriptomic data has identified over 3,000 alternatively spliced genes after mechanical stretch of podocytes as a model of glomerular hypertension.^250^ Podocytes are specialized epithelial cells that wrap around the kidneys’ glomerular capillaries. Changes in capillary pressure and blood flow at the glomerulus generate mechanical strain on podocytes.^251,252^ Podocytes are mechanosensitive because they remodel their cytoskeletons in response to mechanical strain *in vitro* or during *in vivo* hypertension.^250–253^ Similar to our observations in skeletal muscle, mechanical stretching of cultured murine podocytes (over 3 days) induced global changes in alternative splicing landscapes.^250^

The two studies discussed above in the kidney and skeletal muscle are the first evidence that mechanosensitive splicing networks might exist in different cells but might be cell-type- and/or tissue-specific. Thus, future studies should assess the functional consequences of the activation of mechanosensitive splicing networks, and further global transcriptomic studies should be conducted in other cell and tissue contexts where mechanotransduction is important.

### Alternative polyadenylation in cardiac diseases

Alternative polyadenylation is another main RNA processing mechanism that modulates gene expression.^25^ Around 70% of mammalian protein-coding genes can produce transcripts with 3^′^ UTRs of different lengths, which can regulate mRNA localization, stability, and translation rate.^21,254^ Akin to alternative splicing networks, genome-wide trends in the 3^′^ UTR cleavage and polyadenylation are correlated with complex trait heritability and disease heritability in humans.^21,255–257^ Moreover, there is a vast body of research about alternative polyadenylation as a common cellular stress response.^258–260^

Previous work showed that bovine aortic endothelial cells exposed to laminar shear stress expressed a higher proportion of endothelial nitric oxide synthase transcripts with long 3^′^ UTRs,^261^ though this study did not evaluate global changes in alternative polyadenylation. There are, however, two studies on global alternative polyadenylation signatures in cardiac diseases, which involve dysregulation of mechanotransduction as a contributor to disease progression.^262,263^ In this direction, a genome-wide study from Creemers and colleagues compared healthy and dilated cardiomyopathy human hearts and identified heart failure alternative polyadenylation signatures.^248^ In heart failure, transcripts encoding proteins involved in nucleic acid binding, protein binding, and catalytic activity had shortened 3^′^ UTRs, whereas transcripts encoding proteins involved in actin binding, cytoskeletal protein binding, and structural constituents of the cytoskeleton had lengthened 3^′^ UTRs (Figure 4B).^248^ Therefore, while global polyadenylation tail lengths did not follow one trend in heart failure, transcripts with similar cellular functions presented similar trends in polyadenylation tail length.^248^ The authors further established that some of the mRNAs undergoing switches in cleavage site due to dilated cardiomyopathy exhibited different translation efficiency; shortened 3^′^ UTRs correlated with reduced translation and lengthened 3^′^ UTRs correlated with increased translation (Figure 4B).^248^ Similarly, Soetano and colleagues carried out a transcriptomic study in cardiomyocytes isolated from mice undergoing left ventricular hypertrophy (induced by thoracic aortic constriction), revealing a global dysregulation of alternative polyadenylation with predominantly shortened 3^′^ UTRs (Figure 4C).^249^ Genes with the most dramatic shifts toward proximal 3^′^ UTR after thoracic aortic constriction encoded proteins involved in RNA metabolism and splicing, protein ubiquitination and phosphorylation, and intracellular signaling (Figure 4C).^249^

These two studies indicate that alternative polyadenylation might have an important role in regulating changes in gene expression required for pathological cardiac remodeling. However, we still do not know which mechanosensors and mechanotransducers are responsible for triggering the changes in alternative polyadenylation observed by Creemers et al. and Soetano et al.^248,249^ We also do not have evidence to say that the alterations in polyA site selection directly cause changes in protein output; it is highly probable that multiple regulatory layers exist, such as mechanisms involving non-coding RNAs and different RBPs. Moreover, it is important to caution that the trends observed in the discussed studies do not represent a generalizable rule to describe poly(A) tail length and translational output. Transcripts with differential 3^′^ UTRs generated from alternative polyadenylation can exhibit transcript-specific localization and protein-RNA interactions,^264^ which may also modify the transcriptomes and proteomes—this is an additional limitation of what is presented above. Definitely, further work is needed to better understand the underlying mechanism and significance of alternative polyadenylation in cells under mechanical stress. Nevertheless, the existing studies clearly indicate that global changes in 3^′^ UTR length is a cellular response to pathological cardiac remodeling, which is characterized by drastic mechanical changes.

## CONCLUSIONS

Mechanotransduction is critical for individual cell behavior, function, and health as well as for tissue homeostasis under various mechanical stressors. On the other hand, RNA processing is essential for tuning gene expression during all biological processes, including mechanotransduction. Recent studies have only begun to explore the interplay between RNA processing and mechanotransduction. In this review, we have described three emerging areas of research: the functions of RBPs in mechanotransduction, alternative splicing of mechanosensitive proteins, and the role of mechanical forces in regulating RNA processing programs. However, we have also identified some important gaps still present in the field. One major gap is partially due to technological limitations, as until recently, it was very challenging to generate datasets to analyze global alternative polyadenylation. Consequentially, there are only a few studies examining the physiological significance of alternative polyadenylation, and even fewer investigating the connection between alternative polyadenylation and mechanotransduction. The studies reviewed above provide justification for the pursuit of this line of research to characterize global alternative polyadenylation changes in response to mechanical stressors.

Another outstanding gap in the field is the lack of attempts to connect the changes in activity of mechanosensors to the global changes in transcription and RNA processing. In other words, there are a few existing models that map mechanotransduction pathways from what we view as the true start to finish: from the moment when a mechanical signal is received by a cell and transduced through biochemical signaling to the point when it is translated into gene expression changes that are modulated by RNA processing. Our group has begun to do so in skeletal muscle cells, where we have identified a specific mechanosensitive RBP (SRSF4).^93^ Because mechanotransduction is implicated in the homeostasis and function of numerous tissues and organ systems, future work should characterize the transcriptomic and splicing landscapes that are sensitive to specific mechanical stimuli.

Because mechanotransduction is important in human diseases, particularly cardiac pathologies and cancer, and the increased clinical interest in using RNAs and RBPs as therapeutic targets,^265–270^ more research about the relationship between mechanotransduction and RNA processing will permit further development of these novel RNA-based treatments. Of particular interest are HNRNPs, given their roles in tumorigenesis and cardiovascular diseases that we described above. We are hopeful that the future will unveil even more interesting and clinically significant relationships between mechanotransduction and RNA processing.

## Supplementary Material

1

Supplemental information can be found online at https://doi.org/10.1016/j.celrep.2025.116175.

## References

[1] Jaalouk DE, and Lammerding J (2009). Mechanotransduction gone awry. Nat. Rev. Mol. Cell Biol 10, 63–73. 19197333 10.1038/nrm2597 PMC2668954

[2] Di X, Gao X, Peng L, Ai J, Jin X, Qi S, Li H, Wang K, and Luo D (2023). Cellular mechanotransduction in health and diseases: from molecular mechanism to therapeutic targets. Signal Transduct. Target. Ther 8, 282. 37518181 10.1038/s41392-023-01501-9 PMC10387486

[3] Sweeney HL, and Hammers DW (2018). Muscle Contraction. Cold Spring Harb. Perspect. Biol 10, a023200. 29419405 10.1101/cshperspect.a023200 PMC5793755

[4] Discher DE, Mooney DJ, and Zandstra PW (2009). Growth Factors, Matrices, and Forces Combine and Control Stem Cells. Science 324, 1673–1677. 19556500 10.1126/science.1171643 PMC2847855

[5] Hahn C, and Schwartz MA (2009). Mechanotransduction in vascular physiology and atherogenesis. Nat. Rev. Mol. Cell Biol 10, 53–62. 19197332 10.1038/nrm2596 PMC2719300

[6] Wang N, Tytell JD, and Ingber DE (2009). Mechanotransduction at a distance: Mechanically coupling the extracellular matrix with the nucleus. Nat. Rev. Mol. Cell Biol 10, 75–82. 19197334 10.1038/nrm2594

[7] Wozniak MA, and Chen CS (2009). Mechanotransduction in development: a growing role for contractility. Nat. Rev. Mol. Cell Biol 10, 34–43. 19197330 10.1038/nrm2592 PMC2952188

[8] Engler AJ, Sen S, Sweeney HL, and Discher DE (2006). Matrix elasticity directs stem cell lineage specification. Cell 126, 677–689. 16923388 10.1016/j.cell.2006.06.044

[9] Saraswathibhatla A, Indana D, and Chaudhuri O (2023). Cell–extracellular matrix mechanotransduction in 3D. Nat. Rev. Mol. Cell Biol 24, 495–516. 36849594 10.1038/s41580-023-00583-1 PMC10656994

[10] Bansaccal N, Vieugue P, Sarate R, Song Y, Minguijon E, Miroshnikova YA, Zeuschner D, Collin A, Allard J, Engelman D, (2023). The extracellular matrix dictates regional competence for tumour initiation. Nature 623, 828–835. 37968399 10.1038/s41586-023-06740-y PMC7615367

[11] Gilkes DM, Semenza GL, and Wirtz D (2014). Hypoxia and the extracellular matrix: drivers of tumour metastasis. Nat. Rev. Cancer 14, 430–439. 24827502 10.1038/nrc3726 PMC4283800

[12] Wei SC, Fattet L, Tsai JH, Guo Y, Pai VH, Majeski HE, Chen AC, Sah RL, Taylor SS, Engler AJ, and Yang J (2015). Matrix stiffness drives epithelial–mesenchymal transition and tumour metastasis through a TWIST1–G3BP2 mechanotransduction pathway. Nat. Cell Biol 17, 678–688. 25893917 10.1038/ncb3157 PMC4452027

[13] Paszek MJ, Zahir N, Johnson KR, Lakins JN, Rozenberg GI, Gefen A, Reinhart-King CA, Margulies SS, Dembo M, Boettiger D, (2005). Tensional homeostasis and the malignant phenotype. Cancer Cell 8, 241–254. 16169468 10.1016/j.ccr.2005.08.010

[14] Wang JH-C, Thampatty BP, Lin J-S, and Im H-J (2007). Mechanoregulation of gene expression in fibroblasts. Gene 391, 1–15. 17331678 10.1016/j.gene.2007.01.014 PMC2893340

[15] Eroshkin FM, and Zaraisky AG (2017). Mechano-sensitive regulation of gene expression during the embryonic development. genesis 55. 10.1002/dvg.23026 28236362

[16] Copland IB, and Post M (2007). Stretch-activated signaling pathways responsible for early response gene expression in fetal lung epithelial cells. J. Cell. Physiol 210, 133–143. 16998809 10.1002/jcp.20840

[17] Manning KS, and Cooper TA (2017). The roles of RNA processing in translating genotype to phenotype. Nat. Rev. Mol. Cell Biol 18, 102–114. 27847391 10.1038/nrm.2016.139 PMC5544131

[18] Pope SD, and Medzhitov R (2018). Emerging Principles of Gene Expression Programs and Their Regulation. Mol. Cell 71, 389–397. 30075140 10.1016/j.molcel.2018.07.017

[19] Misteli T (2020). The Self-Organizing Genome: Principles of Genome Architecture and Function. Cell 183, 28–45. 32976797 10.1016/j.cell.2020.09.014 PMC7541718

[20] Choquet K, Patop IL, and Churchman LS (2025). The regulation and function of post-transcriptional RNA splicing. Nat. Rev. Genet 26, 378–394. 40217094 10.1038/s41576-025-00836-z

[21] Tian B, and Manley JL (2017). Alternative polyadenylation of mRNA precursors. Nat. Rev. Mol. Cell Biol 18, 18–30. 27677860 10.1038/nrm.2016.116 PMC5483950

[22] Tian B, Hu J, Zhang H, and Lutz CS (2005). A large-scale analysis of mRNA polyadenylation of human and mouse genes. Nucleic Acids Res. 33, 201–212. 15647503 10.1093/nar/gki158 PMC546146

[23] Nakka K, Ghigna C, Gabellini D, and Dilworth FJ (2018). Diversification of the muscle proteome through alternative splicing. Skelet. Muscle 8, 8. 29510724 10.1186/s13395-018-0152-3 PMC5840707

[24] Ule J, and Blencowe BJ (2019). Alternative Splicing Regulatory Networks: Functions, Mechanisms, and Evolution. Mol. Cell 76, 329–345. 31626751 10.1016/j.molcel.2019.09.017

[25] Mitschka S, and Mayr C (2022). Context-specific regulation and function of mRNA alternative polyadenylation. Nat. Rev. Mol. Cell Biol 23, 779–796. 35798852 10.1038/s41580-022-00507-5 PMC9261900

[26] Pandya-Jones A, Bhatt DM, Lin CH, Tong AJ, Smale ST, and Black DL (2013). Splicing kinetics and transcript release from the chromatin compartment limit the rate of Lipid A-induced gene expression. RNA 19, 811–827. 23616639 10.1261/rna.039081.113 PMC3683915

[27] Huang L, Li G, Du C, Jia Y, Yang J, Fan W, Xu YZ, Cheng H, and Zhou Y (2023). The polyA tail facilitates splicing of last introns with weak 3’ splice sites via PABPN1. EMBO Rep. 24, e57128. 37661812 10.15252/embr.202357128 PMC10561182

[28] Cooper TA, Wan L, and Dreyfuss G (2009). RNA and Disease. Cell 136, 777–793. 10.1016/j.cell.2009.02.011 PMC2866189 19239895

[29] Baralle FE, and Giudice J (2017). Alternative splicing as a regulator of development and tissue identity. Nat. Rev. Mol. Cell Biol 18, 437–451. 28488700 10.1038/nrm.2017.27 PMC6839889

[30] Marasco LE, and Kornblihtt AR (2023). The physiology of alternative splicing. Nat. Rev. Mol. Cell Biol 24, 242–254. 36229538 10.1038/s41580-022-00545-z

[31] Ingber DE (2006). Cellular mechanotransduction: putting all the pieces together again. FASEB J. 20, 811–827. 16675838 10.1096/fj.05-5424rev

[32] Janoštiak R, Pataki AC, Brábek J, and Rösel D (2014). Mechanosensors in integrin signaling: the emerging role of p130Cas. Eur. J. Cell Biol 93, 445–454. 25062607 10.1016/j.ejcb.2014.07.002

[33] Hoffman BD, Grashoff C, and Schwartz MA (2011). Dynamic molecular processes mediate cellular mechanotransduction. Nature 475, 316–323. 21776077 10.1038/nature10316 PMC6449687

[34] Coste B, Mathur J, Schmidt M, Earley TJ, Ranade S, Petrus MJ, Dubin AE, and Patapoutian A (2010). Piezo1 and Piezo2 are essential components of distinct mechanically activated cation channels. Science 330, 55–60. 20813920 10.1126/science.1193270 PMC3062430

[35] Honoré E (2007). The neuronal background K2P channels: Focus on TREK1. Nat. Rev. Neurosci 8, 251–261. 10.1038/nrn2117. 17375039

[36] Reitsma S, Slaaf DW, Vink H, Van Zandvoort MAMJ, and Oude Egbrink MGA (2007). The endothelial glycocalyx: Composition, functions, and visualization. Pflugers Arch. 454, 345–359. 10.1007/s00424-007-0212-8. 17256154 PMC1915585

[37] Caprara GA, and Peng AW (2022). Mechanotransduction in mammalian sensory hair cells. Mol. Cell. Neurosci 120, 103706. 35218890 10.1016/j.mcn.2022.103706 PMC9177625

[38] Tarbell JM, and Ebong EE (2008). The endothelial glycocalyx: a mechano-sensor and -transducer. Sci. Signal 1, pt8. 18840877 10.1126/scisignal.140pt8

[39] Roca-Cusachs P, del Rio A, Puklin-Faucher E, Gauthier NC, Biais N, and Sheetz MP (2013). Integrin-dependent force transmission to the extracellular matrix by α-actinin triggers adhesion maturation. Proc. Natl. Acad. Sci. USA 110, E1361–E1370. 23515331 10.1073/pnas.1220723110 PMC3625291

[40] Ross TD, Coon BG, Yun S, Baeyens N, Tanaka K, Ouyang M, and Schwartz MA (2013). Integrins in mechanotransduction. Curr. Opin. Cell Biol 25, 613–618. 23797029 10.1016/j.ceb.2013.05.006 PMC3757118

[41] Schiller HB, Hermann MR, Polleux J, Vignaud T, Zanivan S, Friedel CC, Sun Z, Raducanu A, Gottschalk KE, Théry M, (2013). β 1 - And α v -class integrins cooperate to regulate myosin II during rigidity sensing of fibronectin-based microenvironments. Nat. Cell Biol 15, 625–636. 23708002 10.1038/ncb2747

[42] Strohmeyer N, Bharadwaj M, Costell M, Fässler R, and Müller DJ (2017). Fibronectin-bound α5β1 integrins sense load and signal to reinforce adhesion in less than a second. Nat. Mater 16, 1262–1270. 29115292 10.1038/nmat5023

[43] Alenghat FJ, and Ingber DE (2002). Mechanotransduction: all signals point to cytoskeleton, matrix, and integrins. Sci. STKE 2002, pe6. 11842240 10.1126/stke.2002.119.pe6

[44] Müller P, Langenbach A, Kaminski A, and Rychly J (2013). Modulating the Actin Cytoskeleton Affects Mechanically Induced Signal Transduction and Differentiation in Mesenchymal Stem Cells. PLoS One 8, e71283. 23923061 10.1371/journal.pone.0071283 PMC3726577

[45] Seetharaman S, Vianay B, Roca V, Farrugia AJ, De Pascalis C, Boëda B, Dingli F, Loew D, Vassilopoulos S, Bershadsky A, (2022). Microtubules tune mechanosensitive cell responses. Nat. Mater 21, 366–377. 34663953 10.1038/s41563-021-01108-x

[46] Martineau LC, and Gardiner PF (2001). Insight into skeletal muscle mechanotransduction: MAPK activation is quantitatively related to tension. J. Appl. Physiol 91, 693–702. 11457783 10.1152/jappl.2001.91.2.693

[47] Hishikawa K, Oemar BS, Yang Z, and Lüscher TF (1997). Pulsatile stretch stimulates superoxide production and activates nuclear factor-κb in human coronary smooth muscle. Circ. Res 81, 797–803. 9351451 10.1161/01.res.81.5.797

[48] Khachigian LM, Resnick N, Gimbrone MA, and Collins T (1995). Nuclear factor-kappa B interacts functionally with the platelet-derived growth factor B-chain shear-stress response element in vascular endothelial cells exposed to fluid shear stress. J. Clin. Investig 96, 1169–1175. 7635955 10.1172/JCI118106 PMC185309

[49] Calvo F, Ege N, Grande-Garcia A, Hooper S, Jenkins RP, Chaudhry SI, Harrington K, Williamson P, Moeendarbary E, Charras G, and Sahai E (2013). Mechanotransduction and YAP-dependent matrix remodelling is required for the generation and maintenance of cancer-associated fibroblasts. Nat. Cell Biol 15, 637–646. 23708000 10.1038/ncb2756 PMC3836234

[50] Dupont S, Morsut L, Aragona M, Enzo E, Giulitti S, Cordenonsi M, Zanconato F, Le Digabel J, Forcato M, Bicciato S, (2011). Role of YAP/TAZ in mechanotransduction. Nature 474, 179–183. 21654799 10.1038/nature10137

[51] Agarwal P, and Zaidel-Bar R (2021). Mechanosensing in embryogenesis. Curr. Opin. Cell Biol 68, 1–9. 32898827 10.1016/j.ceb.2020.08.007

[52] Tschumperlin DJ, Ligresti G, Hilscher MB, and Shah VH (2018). Mechanosensing and fibrosis. J. Clin. Investig 128, 74–84. 29293092 10.1172/JCI93561 PMC5749510

[53] Bergmann MW, and De Windt LJ (2007). Linking Cardiac Mechanosensing at the Sarcomere M-Band, Nuclear Factor κB Signaling, and Cardiac Remodeling. Hypertension 49, 1225–1227. 17404179 10.1161/HYPERTENSIONAHA.107.089052

[54] Dupont S, and Wickström SA (2022). Mechanical regulation of chromatin and transcription. Nat. Rev. Genet 23, 624–643. 35606569 10.1038/s41576-022-00493-6

[55] Tajik A, Zhang Y, Wei F, Sun J, Jia Q, Zhou W, Singh R, Khanna N, Belmont AS, and Wang N (2016). Transcription upregulation via force-induced direct stretching of chromatin. Nat. Mater 15, 1287–1296. 27548707 10.1038/nmat4729 PMC5121013

[56] Chambliss AB, Khatau SB, Erdenberger N, Robinson DK, Hodzic D, Longmore GD, and Wirtz D (2013). The LINC-anchored actin cap connects the extracellular milieu to the nucleus for ultrafast mechanotransduction. Sci. Rep 3, 1087. 23336069 10.1038/srep01087 PMC3548190

[57] Shiu JY, Aires L, Lin Z, and Vogel V (2018). Nanopillar force measurements reveal actin-cap-mediated YAP mechanotransduction. Nat. Cell Biol 20, 262–271. 29403039 10.1038/s41556-017-0030-y

[58] Kirby TJ, and Lammerding J (2018). Emerging views of the nucleus as a cellular mechanosensor. Nat. Cell Biol 20, 373–381. 10.1038/s41556-018-0038-y. 29467443 PMC6440800

[59] Gebauer F, Schwarzl T, Valcárcel J, and Hentze MW (2021). RNA-binding proteins in human genetic disease. Nat. Rev. Genet 22, 185–198. 33235359 10.1038/s41576-020-00302-y

[60] Van Nostrand EL, Freese P, Pratt GA, Wang X, Wei X, Xiao R, Blue SM, Chen JY, Cody NAL, Dominguez D, (2020). A large-scale binding and functional map of human RNA-binding proteins. Nature 583, 711–719. 32728246 10.1038/s41586-020-2077-3 PMC7410833

[61] Neve J, Patel R, Wang Z, Louey A, and Furger AM (2017). Cleavage and polyadenylation: Ending the message expands gene regulation. RNA Biol. 14, 865–890. 28453393 10.1080/15476286.2017.1306171 PMC5546720

[62] Harpster MH, Bandyopadhyay S, Thomas DP, Ivanov PS, Keele JA, Pineguina N, Gao B, Amarendran V, Gomelsky M, McCormick RJ, and Stayton MM (2006). Earliest changes in the left ventricular transcriptome post-myocardial infarction. Mamm. Genome 17, 701–715. 16845475 10.1007/s00335-005-0120-1

[63] Barth AS, Kuner R, Buness A, Ruschhaupt M, Merk S, Zwermann L, Kääb S, Kreuzer E, Steinbeck G, Mansmann U, (2006). Identification of a Common Gene Expression Signature in Dilated Cardiomyopathy Across Independent Microarray Studies. J. Am. Coll. Cardiol 48, 1610–1617. 17045896 10.1016/j.jacc.2006.07.026

[64] Colak D, Kaya N, Al-Zahrani J, Al Bakheet A, Muiya P, Andres E, Quackenbush J, and Dzimiri N (2009). Left ventricular global transcriptional profiling in human end-stage dilated cardiomyopathy. Genomics 94, 20–31. 19332114 10.1016/j.ygeno.2009.03.003 PMC4152850

[65] Rowell J, Koitabashi N, Kass DA, and Barth AS (2014). Dynamic gene expression patterns in animal models of early and late heart failure reveal biphasic-bidirectional transcriptional activation of signaling pathways. Physiol. Genomics 46, 779–787. 25159852 10.1152/physiolgenomics.00054.2014 PMC4200378

[66] Martino F, Varadarajan NM, Perestrelo AR, Hejret V, Durikova H, Vukic D, Horvath V, Cavalieri F, Caruso F, Albihlal WS, (2022). The mechanical regulation of RNA binding protein hnRNPC in the failing heart. Sci. Transl. Med 14, eabo5715. 36417487 10.1126/scitranslmed.abo5715

[67] Chaudhury A, Chander P, and Howe PH (2010). Heterogeneous nuclear ribonucleoproteins (hnRNPs) in cellular processes: Focus on hnRNP E1’s multifunctional regulatory roles. RNA 16, 1449–1462. 20584894 10.1261/rna.2254110 PMC2905745

[68] Geuens T, Bouhy D, and Timmerman V (2016). The hnRNP family: insights into their role in health and disease. Hum. Genet 135, 851–867. 27215579 10.1007/s00439-016-1683-5 PMC4947485

[69] Qi Y, Yu J, Han W, Fan X, Qian H, Wei H, Tsai YHS, Zhao J, Zhang W, Liu Q, (2016). A splicing isoform of TEAD4 attenuates the Hippo–YAP signalling to inhibit tumour proliferation. Nat. Commun 7, ncomms11840. 10.1038/ncomms11840 PMC4909989 27291620

[70] Gao C, Ren S, Lee JH, Qiu J, Chapski DJ, Rau CD, Zhou Y, Abdellatif M, Nakano A, Vondriska TM, (2016). RBFox1-mediated RNA splicing regulates cardiac hypertrophy and heart failure. J. Clin. Investig 126, 195–206. 26619120 10.1172/JCI84015 PMC4701548

[71] Zorn P, Calvo Sánchez J, Alakhras T, Schreier B, Gekle M, Hüttelmaier S, and Köhn M (2024). Rbfox1 controls alternative splicing of focal adhesion genes in cardiac muscle cells. J. Mol. Cell Biol 16, mjae003. 10.1093/jmcb/mjae003. 38253401 PMC11216089

[72] Vrbský J, Vinarský V, Perestrelo AR, De La Cruz JO, Martino F, Pompeiano A, Izzi V, Hlinomaz O, Rotrekl V, Sudol M, (2021). Evidence for discrete modes of YAP1 signaling via mRNA splice isoforms in development and diseases. Genomics 113, 1349–1365. 33713822 10.1016/j.ygeno.2021.03.009

[73] Nardone G, Oliver-De La Cruz J, Vrbsky J, Martini C, Pribyl J, Skládal P, Pe sl M, Caluori G, Pagliari S, Martino F, (2017). YAP regulates cell mechanics by controlling focal adhesion assembly. Nat. Commun 8, 15321. 28504269 10.1038/ncomms15321 PMC5440673

[74] Lin PK, and Davis GE (2023). Extracellular Matrix Remodeling in Vascular Disease: Defining Its Regulators and Pathological Influence. Arterioscler. Thromb. Vasc. Biol 43, 1599–1616. 37409533 10.1161/ATVBAHA.123.318237 PMC10527588

[75] Huang J, Zhang L, Wan D, Zhou L, Zheng S, Lin S, and Qiao Y (2021). Extracellular matrix and its therapeutic potential for cancer treatment. Signal Transduct. Target. Ther 6, 153. 33888679 10.1038/s41392-021-00544-0 PMC8062524

[76] Kim HE, Dalal SS, Young E, Legato MJ, Weisfeldt ML, and D’Armiento J (2000). Disruption of the myocardial extracellular matrix leads to cardiac dysfunction. J. Clin. Investig 106, 857–866. 11018073 10.1172/JCI8040 PMC517818

[77] Chakraborty S, Njah K, Pobbati AV, Lim YB, Raju A, Lakshmanan M, Tergaonkar V, Lim CT, and Hong W (2017). Agrin as a Mechanotransduction Signal Regulating YAP through the Hippo Pathway. Cell Rep. 18, 2464–2479. 28273460 10.1016/j.celrep.2017.02.041

[78] Fu M, Hu Y, Lan T, Guan KL, Luo T, and Luo M (2022). The Hippo signalling pathway and its implications in human health and diseases. Signal Transduct. Target. Ther 7, 376. 36347846 10.1038/s41392-022-01191-9 PMC9643504

[79] Kwon Y, Vinayagam A, Sun X, Dephoure N, Gygi SP, Hong P, and Perrimon N (2013). The Hippo Signaling Pathway Interactome. Science 342, 737–740. 24114784 10.1126/science.1243971 PMC3951131

[80] Li Z, Chen S, Cui H, Li X, Chen D, Hao W, Wang J, Li Z, Zheng Z, Zhang Z, and Liu H (2021). Tenascin-C-mediated suppression of extracellular matrix adhesion force promotes entheseal new bone formation through activation of Hippo signalling in ankylosing spondylitis. Ann. Rheum. Dis 80, 891–902. 33858850 10.1136/annrheumdis-2021-220002 PMC8237173

[81] Ma S, Meng Z, Chen R, and Guan K-L (2018). The Hippo Pathway: Biology and Pathophysiology. Annu Rev Biochem 88, 577–604. 10.1146/annurev-biochem-013118. 30566373

[82] Meng Z, Qiu Y, Lin KC, Kumar A, Placone JK, Fang C, Wang KC, Lu S, Pan M, Hong AW, (2018). RAP2 mediates mechanoresponses of the Hippo pathway. Nature 560, 655–660. 30135582 10.1038/s41586-018-0444-0 PMC6128698

[83] Pereira D, Richert A, Medjkane S, Hénon S, and Weitzman JB (2020). Cell geometry and the cytoskeleton impact the nucleo-cytoplasmic localisation of the SMYD3 methyltransferase. Sci. Rep 10, 20598. 33244033 10.1038/s41598-020-75833-9 PMC7691988

[84] Roux E, Bougaran P, Dufourcq P, and Couffinhal T (2020). Fluid Shear Stress Sensing by the Endothelial Layer. Front. Physiol 11, 861. 10.3389/fphys.2020.00861. 32848833 PMC7396610

[85] Wada K-I, Itoga K, Okano T, Yonemura S, and Sasaki H (2011). Hippo pathway regulation by cell morphology and stress fibers. Development 138, 3907–3914. 21831922 10.1242/dev.070987

[86] Wang Y, Chen D, Qian H, Tsai YS, Shao S, Liu Q, Dominguez D, and Wang Z (2014). The Splicing Factor RBM4 Controls Apoptosis, Proliferation, and Migration to Suppress Tumor Progression. Cancer Cell 26, 374–389. 25203323 10.1016/j.ccr.2014.07.010 PMC4159621

[87] Lin J-C, Lee YC, Liang YC, Fann YC, Johnson KR, and Lin YJ (2017). The impact of the RBM4-initiated splicing cascade on modulating the carcinogenic signature of colorectal cancer cells. Sci. Rep 7, 44204. 28276498 10.1038/srep44204 PMC5343574

[88] Chen L, Zhang W, Chen D, Yang Q, Sun S, Dai Z, Li Z, Liang X, Chen C, Jiao Y, (2023). RBM4 dictates ESCC cell fate switch from cellular senescence to glutamine-addiction survival through inhibiting LKB1-AMPK-axis. Signal Transduct. Target. Ther 8, 159. 37080995 10.1038/s41392-023-01367-x PMC10119322

[89] Duan Y, Liu S, Wang J, Yang K, Xu J, Wang Q, Liu J, Hao J, Cui X, Tan Y, (2024). Overexpression of RBM4 promotes acute myeloid leukemia cell differentiation by regulating alternative splicing of TFEB. J. Biol. Chem 300, 107729. 39214303 10.1016/j.jbc.2024.107729 PMC11467665

[90] Lin J-C, Hsu M, and Tarn W-Y (2007). Cell stress modulates the function of splicing regulatory protein RBM4 in translation control. Proc. Natl. Acad. Sci. USA 104, 2235–2240. 17284590 10.1073/pnas.0611015104 PMC1893002

[91] Lin J-C, and Tarn W-Y (2009). RNA-binding Motif Protein 4 Translocates to Cytoplasmic Granules and Suppresses Translation via Argo- naute2 during Muscle Cell Differentiation. J. Biol. Chem 284, 34658–34665. 19801630 10.1074/jbc.M109.032946 PMC2787328

[92] Cao W, Jamison SF, and Garcia-Blanco MA (1997). Both phosphorylation and dephosphorylation of ASF/SF2 are required for pre-mRNA splicing in vitro. RNA 3, 1456–1467. 9404896 PMC1369586

[93] Hinkle ER, Blue RE, Tsai YH, Combs M, Davi J, Coffey AR, Boriek AM, Taylor JM, Parker JS, and Giudice J (2022). Stretching muscle cells induces transcriptional and splicing transitions and changes in SR proteins. Commun. Biol 5, 987. 36123433 10.1038/s42003-022-03915-7 PMC9485123

[94] Bordeleau F, Califano JP, Negrón Abril YL, Mason BN, LaValley DJ, Shin SJ, Weiss RS, and Reinhart-King CA (2015). Tissue stiffness regulates serine/arginine-rich protein-mediated splicing of the extra domain B-fibronectin isoform in tumors. Proc. Natl. Acad. Sci. USA 112, 8314–8319. 26106154 10.1073/pnas.1505421112 PMC4500261

[95] Hauptmann S, Zardi L, Siri A, Carnemolla B, Borsi L, Castellucci M, Klosterhalfen B, Hartung P, Weis J, and Stöcker G (1995). Extracellular matrix proteins in colorectal carcinomas. Expression of tenascin and fibronectin isoforms. Lab. Invest 73, 172–182. 7543628

[96] Scarpino S, Stoppacciaro A, Pellegrini C, Marzullo A, Zardi L, Tartaglia F, Viale G, and Ruco LP (1999). Expression of EDA/EDB isoforms of fibronectin in papillary carcinoma of the thyroid. J. Pathol 188, 163–167. 10398159 10.1002/(SICI)1096-9896(199906)188:2<163::AID-PATH335>3.0.CO;2-1

[97] Astrof S, Crowley D, George EL, Fukuda T, Sekiguchi K, Hanahan D, and Hynes RO (2004). Direct Test of Potential Roles of EIIIA and EIIIB Alternatively Spliced Segments of Fibronectin in Physiological and Tumor Angiogenesis. Mol. Cell Biol 24, 8662–8670. 15367684 10.1128/MCB.24.19.8662-8670.2004 PMC516752

[98] Srebrow A, Blaustein M, and Kornblihtt AR (2002). Regulation of fibronectin alternative splicing by a basement membrane-like extracellular matrix. FEBS Lett. 514, 285–289. 11943167 10.1016/s0014-5793(02)02382-7

[99] Anczuków O, Akerman M, Cléry A, Wu J, Shen C, Shirole NH, Raimer A, Sun S, Jensen MA, Hua Y, (2015). SRSF1-Regulated Alternative Splicing in Breast Cancer. Mol. Cell 60, 105–117. 26431027 10.1016/j.molcel.2015.09.005 PMC4597910

[100] Urbanski LM, Leclair N, and Anczuków O (2018). Alternative-splicing defects in cancer: Splicing regulators and their downstream targets, guiding the way to novel cancer therapeutics. Wiley Interdiscip. Rev. RNA 9, e1476. 29693319 10.1002/wrna.1476 PMC6002934

[101] Blaustein M, Pelisch F, Tanos T, Muñoz MJ, Wengier D, Quadrana L, Sanford JR, Muschietti JP, Kornblihtt AR, Cáceres JF, (2005). Concerted regulation of nuclear and cytoplasmic activities of SR proteins by AKT. Nat. Struct. Mol. Biol 12, 1037–1044. 16299516 10.1038/nsmb1020

[102] Patel NA, Kaneko S, Apostolatos HS, Bae SS, Watson JE, Davidowitz K, Chappell DS, Birnbaum MJ, Cheng JQ, and Cooper DR (2005). Molecular and genetic studies imply Akt-mediated signaling promotes protein kinase CbetaII alternative splicing via phosphorylation of serine/arginine-rich splicing factor SRp40. J. Biol. Chem 280, 14302–14309. 15684423 10.1074/jbc.M411485200

[103] Mouw JK, Yui Y, Damiano L, Bainer RO, Lakins JN, Acerbi I, Ou G, Wijekoon AC, Levental KR, Gilbert PM, (2014). Tissue mechanics modulate microRNA-dependent PTEN expression to regulate malignant progression. Nat. Med 20, 360–367. 24633304 10.1038/nm.3497 PMC3981899

[104] Wu B, Liu DA, Guan L, Myint PK, Chin L, Dang H, Xu Y, Ren J, Li T, Yu Z, (2023). Stiff matrix induces exosome secretion to promote tumour growth. Nat. Cell Biol 25, 415–424. 36797475 10.1038/s41556-023-01092-1 PMC10351222

[105] Phanish MK, Heidebrecht F, Nabi ME, Shah N, Niculescu-Duvaz I, and Dockrell MEC (2015). The Regulation of TGFβ1 Induced Fibronectin EDA Exon Alternative Splicing in Human Renal Proximal Tubule Epithelial Cells. J. Cell. Physiol 230, 286–295. 24962218 10.1002/jcp.24703

[106] Conboy JG (2017). Developmental regulation of RNA processing by Rbfox proteins. WIREs RNA 8. 10.1002/wrna.1398 PMC5315656 27748060

[107] Jin Y, Suzuki H, Maegawa S, Endo H, Sugano S, Hashimoto K, Yasuda K, and Inoue K (2003). A vertebrate RNA-binding protein Fox-1 regulates tissue-specific splicing via the pentanucleotide GCAUG. EMBO J. 22, 905–912. 12574126 10.1093/emboj/cdg089 PMC145449

[108] Lambert N, Robertson A, Jangi M, McGeary S, Sharp PA, and Burge CB (2014). RNA Bind-n-Seq: Quantitative Assessment of the Sequence and Structural Binding Specificity of RNA Binding Proteins. Mol. Cell 54, 887–900. 24837674 10.1016/j.molcel.2014.04.016 PMC4142047

[109] Zhang C, Zhang Z, Castle J, Sun S, Johnson J, Krainer AR, and Zhang MQ (2008). Defining the regulatory network of the tissue-specific splicing factors Fox-1 and Fox-2. Genes Dev. 22, 2550–2563. 18794351 10.1101/gad.1703108 PMC2546699

[110] Kalsotra A, Xiao X, Ward AJ, Castle JC, Johnson JM, Burge CB, and Cooper TA (2008). A postnatal switch of CELF and MBNL proteins reprograms alternative splicing in the developing heart. Proc. Natl. Acad. Sci. USA 105, 20333–20338. 19075228 10.1073/pnas.0809045105 PMC2629332

[111] Frese KS, Meder B, Keller A, Just S, Haas J, Vogel B, Fischer S, Backes C, Matzas M, Köhler D, (2015). RNA splicing regulated by RBFOX1 is essential for cardiac function in zebrafish. J. Cell Sci 128, 3030–3040. 10.1242/jcs.166850. 26116573 PMC4541041

[112] Sastry SK, and Burridge K (2000). Focal Adhesions: A Nexus for Intracellular Signaling and Cytoskeletal Dynamics. Exp. Cell Res 261, 25–36. 11082272 10.1006/excr.2000.5043

[113] Burridge K, and Chrzanowska-Wodnicka M (1996). FOCAL ADHESIONS, CONTRACTILITY, AND SIGNALING. Annu. Rev. Cell Dev. Biol 12, 463–518. 8970735 10.1146/annurev.cellbio.12.1.463

[114] Calderwood DA, Shattil SJ, and Ginsberg MH (2000). Integrins and Actin Filaments: Reciprocal Regulation of Cell Adhesion and Signaling. J. Biol. Chem 275, 22607–22610. 10801899 10.1074/jbc.R900037199

[115] Feramisco JR, Smart JE, Burridge K, Helfman DM, and Thomas GP (1982). Co-existence of vinculin and a vinculin-like protein of higher molecular weight in smooth muscle. J. Biol. Chem 257, 11024–11031. 6809764

[116] Glukhova MA, Kabakov AE, Belkin AM, Frid MG, Ornatsky OI, Zhidkova NI, and Koteliansky VE (1986). Meta-vinculin distribution in adult human tissues and cultured cells. FEBS Lett. 207, 139–141. 3095141 10.1016/0014-5793(86)80027-8

[117] Belkin AM, Ornatsky OI, Kabakov AE, Glukhova MA, and Koteliansky VE (1988). Diversity of vinculin/meta-vinculin in human tissues and cultivated cells. Expression of muscle specific variants of vinculin in human aorta smooth muscle cells. J. Biol. Chem 263, 6631–6635. 3129429

[118] Dumbauld DW, Lee TT, Singh A, Scrimgeour J, Gersbach CA, Zamir EA, Fu J, Chen CS, Curtis JE, Craig SW, and García AJ (2013). How vinculin regulates force transmission. Proc. Natl. Acad. Sci. USA 110, 9788–9793. 23716647 10.1073/pnas.1216209110 PMC3683711

[119] Atherton P, Stutchbury B, Wang DY, Jethwa D, Tsang R, Meiler-Rodriguez E, Wang P, Bate N, Zent R, Barsukov IL, (2015). Vinculin controls talin engagement with the actomyosin machinery. Nat. Commun 6, 10038. 26634421 10.1038/ncomms10038 PMC4686655

[120] Tseng P-L, Sun W, Salem A, Alaklobie M, Macfarlane SC, Gad AKB, Collins MO, and Erdmann KS (2025). Mechanical control of the alternative splicing factor PTBP1 regulates extracellular matrix stiffness induced proliferation and cell spreading. iScience 28, 112273. 40241749 10.1016/j.isci.2025.112273 PMC12002664

[121] Keppetipola N, Sharma S, Li Q, and Black DL (2012). Neuronal regulation of pre-mRNA splicing by polypyrimidine tract binding proteins, PTBP1 and PTBP2. Crit. Rev. Biochem. Mol. Biol 47, 360–378. 22655688 10.3109/10409238.2012.691456 PMC3422667

[122] Yeh Y-C, Ling J-Y, Chen W-C, Lin H-H, and Tang M-J (2017). Mechanotransduction of matrix stiffness in regulation of focal adhesion size and number: reciprocal regulation of caveolin-1 and β1 integrin. Sci. Rep 7, 15008. 29118431 10.1038/s41598-017-14932-6 PMC5678369

[123] Du J, Chen X, Liang X, Zhang G, Xu J, He L, Zhan Q, Feng XQ, Chien S, and Yang C (2011). Integrin activation and internalization on soft ECM as a mechanism of induction of stem cell differentiation by ECM elasticity. Proc. Natl. Acad. Sci. USA 108, 9466–9471. 21593411 10.1073/pnas.1106467108 PMC3111285

[124] To WS, and Midwood KS (2011). Plasma and cellular fibronectin: distinct and independent functions during tissue repair. Fibrogenesis Tissue Repair 4, 21. 21923916 10.1186/1755-1536-4-21 PMC3182887

[125] Patten J, and Wang K (2021). Fibronectin in development and wound healing. Adv. Drug Deliv. Rev 170, 353–368. 32961203 10.1016/j.addr.2020.09.005

[126] Elosegui-Artola A, Bazellières E, Allen MD, Andreu I, Oria R, Sunyer R, Gomm JJ, Marshall JF, Jones JL, Trepat X, and Roca-Cusachs P (2014). Rigidity sensing and adaptation through regulation of integrin types. Nat. Mater 13, 631–637. 24793358 10.1038/nmat3960 PMC4031069

[127] Kornblihtt AR, Umezawa K, Vibe-Pedersen K, and Baralle FE (1985). Primary structure of human fibronectin: differential splicing may generate at least 10 polypeptides from a single gene. EMBO J. 4, 1755–1759. 2992939 10.1002/j.1460-2075.1985.tb03847.x PMC554414

[128] Kornblihtt AR, Vibe-Pedersen K, and Baralle FE (1984). Human fibronectin: cell specific alternative mRNA splicing generates polypeptide chains dfffering in the number of internal repeats. Nucleic Acids Res. 12, 5853–5868. 6462919 10.1093/nar/12.14.5853 PMC320036

[129] Kornblihtt AR, Pesce CG, Alonso CR, Cramer P, Srebrow A, Werbajh S, and Muro AF (1996). The fibronectin gene as a model for splicing and transcription studies. FASEB J. 10, 248–257. 8641558 10.1096/fasebj.10.2.8641558

[130] Schwarzbauer JE (1991). Alternative splicing of fibronectin: Three variants, three functions. Bioessays 13, 527–533. 1755828 10.1002/bies.950131006

[131] Murphy PA, and Hynes RO (2014). Alternative Splicing of Endothelial Fibronectin Is Induced by Disturbed Hemodynamics and Protects Against Hemorrhage of the Vessel Wall. Arterioscler. Thromb. Vasc. Biol 34, 2042–2050. 24903094 10.1161/ATVBAHA.114.303879 PMC4140979

[132] Murphy PA, Jailkhani N, Nicholas SA, Del Rosario AM, Balsbaugh JL, Begum S, Kimble A, and Hynes RO (2021). Alternative Splicing of FN (Fibronectin) Regulates the Composition of the Arterial Wall Under Low Flow. Arterioscler. Thromb. Vasc. Biol 41, e18–e32. 33207933 10.1161/ATVBAHA.120.314013 PMC8428803

[133] Astrof S, Crowley D, and Hynes RO (2007). Multiple cardiovascular defects caused by the absence of alternatively spliced segments of fibronectin. Dev. Biol 311, 11–24. 17706958 10.1016/j.ydbio.2007.07.005 PMC2080666

[134] Franco-Valls H, Tusquets-Uxó E, Sala L, Val M, Peña R, Iaconcig A, Villarino Á, Jiménez-Arriola M, Massó P, Trincado JL, (2023). Formation of an invasion-permissive matrix requires TGFβ/ SNAIL1-regulated alternative splicing of fibronectin. Breast Cancer Res. 25, 143. 37964360 10.1186/s13058-023-01736-y PMC10647173

[135] Hooper AT, Marquette K, Chang CPB, Golas J, Jain S, Lam MH, Guffroy M, Leal M, Falahatpisheh H, Mathur D, (2022). Anti-Extra Domain B Splice Variant of Fibronectin Antibody-Drug Conjugate Eliminates Tumors with Enhanced Efficacy When Combined with Checkpoint Blockade. Mol. Cancer Ther 21, 1462–1472. 35793468 10.1158/1535-7163.MCT-22-0099 PMC9446899

[136] Phanish MK, Heidebrecht F, Jackson M, Rigo F, and Dockrell MEC (2024). Targeting alternative splicing of fibronectin in human renal proximal tubule epithelial cells with antisense oligonucleotides to reduce EDA+ fibronectin production and block an autocrine loop that drives renal fibrosis. Exp. Cell Res 442, 114186. 39098465 10.1016/j.yexcr.2024.114186

[137] Liao K-C, Chuo V, Fagg WS, Modahl CM, Widen S, and Garcia-Blanco MA (2021). The RNA binding protein Quaking represses splicing of the Fibronectin EDA exon and downregulates the interferon response. Nucleic Acids Res. 49, 10034–10045. 34428287 10.1093/nar/gkab732 PMC8464043

[138] Arriagada C, Lin E, Schonning M, and Astrof S (2025). Mesodermal fibronectin controls cell shape, polarity, and mechanotransduction in the second heart field during cardiac outflow tract development. Dev. Cell 60, 62–84.e7. 10.1016/j.devcel.2024.09.017. 39413783 PMC11706711

[139] Schwarzbauer JE, Patel RS, Fonda D, and Hynes RO (1987). Multiple sites of alternative splicing of the rat fibronectin gene transcript. EMBO J. 6, 2573–2580. 2445560 10.1002/j.1460-2075.1987.tb02547.x PMC553677

[140] Norton PA, and Hynes RO (1987). Alternative Splicing of Chicken Fibronectin in Embryos and in Normal and Transformed Cells. Mol. Cell Biol 7, 4297–4307. 2830487 10.1128/mcb.7.12.4297-4307.1987 PMC368112

[141] Sekiguchi K, Klos AM, Kurachi K, Yoshitake S, and Hakomori S (1986). Human liver fibronectin complementary DNAs: identification of two different messenger RNAs possibly encoding the .alpha. and .beta. subunits of plasma fibronectin. Biochemistry 25, 4936–4941. 3021206 10.1021/bi00365a032

[142] White ES, Baralle FE, and Muro AF (2008). New insights into form and function of fibronectin splice variants. J. Pathol 216, 1–14. 18680111 10.1002/path.2388 PMC4630009

[143] Pankov R, and Yamada KM (2002). Fibronectin at a glance. J. Cell Sci 115, 3861–3863. 12244123 10.1242/jcs.00059

[144] Liao Y-F, Gotwals PJ, Koteliansky VE, Sheppard D, and Van De Water L (2002). The EIIIA Segment of Fibronectin Is a Ligand for Integrins α9β1 and α4β1Providing a Novel Mechanism for Regulating Cell Adhesion by Alternative Splicing. J. Biol. Chem 277, 14467–14474. 11839764 10.1074/jbc.M201100200

[145] Ffrench-Constant C, Van de Water L, Dvorak HF, and Hynes RO (1989). Reappearance of an embryonic pattern of fibronectin splicing during wound healing in the adult rat. J. Cell Biol 109, 903–914. 2760116 10.1083/jcb.109.2.903 PMC2115730

[146] George EL, Georges-Labouesse EN, Patel-King RS, Rayburn H, and Hynes RO (1993). Defects in mesoderm, neural tube and vascular development in mouse embryos lacking fibronectin. Development 119, 1079–1091. 8306876 10.1242/dev.119.4.1079

[147] Peters JH, and Hynes RO (1996). Fibronectin Isoform Distribution in the Mouse I. The Alternatively Spliced EIIIB, EIIIA, and V Segments Show Widespread Codistribution in the Developing Mouse Embryo. Cell Adhes. Commun 4, 103–125. 8937746 10.3109/15419069609010766

[148] Peters JH, Loredo GA, Chen G, Maunder R, Hahn TJ, Willits NH, and Hynes RO (2003). Plasma levels of fibronectin bearing the alternatively spliced EIIIB segment are increased after major trauma. J. Lab. Clin. Med 141, 401–410. 12819638 10.1016/S0022-2143(03)00042-8

[149] Peters JH, Maunder RJ, Woolf AD, Cochrane CG, and Ginsberg MH (1989). Elevated plasma levels of ED1+ (‘cellular’) fibronectin in patients with vascular injury. J. Lab. Clin. Med 113, 586–597. 2715681

[150] Chang M-L, Chen J-C, Alonso CR, Kornblihtt AR, and Bissell DM (2004). Regulation of fibronectin splicing in sinusoidal endothelial cells from normal or injured liver. Proc. Natl. Acad. Sci. USA 101, 18093–18098. 15604136 10.1073/pnas.0408439102 PMC539814

[151] Brown LF, Dubin D, Lavigne L, Logan B, Dvorak HF, and Van de Water L (1993). Macrophages and fibroblasts express embryonic fibronectins during cutaneous wound healing. Am. J. Pathol 142, 793–801. 8456940 PMC1886786

[152] Barnes JL, Hastings RR, and De la Garza MA (1994). Sequential expression of cellular fibronectin by platelets, macrophages, and mesangial cells in proliferative glomerulonephritis. Am. J. Pathol 145, 585–597. 8080041 PMC1890339

[153] Kuhn C, Boldt J, King TE Jr., Crouch E, Vartio T, and McDonald JA (1989). An immunohistochemical study of architectural remodeling and connective tissue synthesis in pulmonary fibrosis. Am. Rev. Respir. Dis 140, 1693–1703. 2604297 10.1164/ajrccm/140.6.1693

[154] Peters JH, Chen GE, and Hynes RO (1996). Fibronectin isoform distribution in the mouse. II. Differential distribution of the alternatively spliced EIIIB, EIIIA, and V segments in the adult mouse. Cell Adhes. Commun 4, 127–148. 8937747 10.3109/15419069609010767

[155] Gehris AL, Oberlender SA, Shepley KJ, Tuan RS, and Bennett VD (1996). Fibronectin mRNA alternative splicing is temporally and spatially regulated during chondrogenesis in vivo and in vitro. Dev. Dyn 206, 219–230. 8725289 10.1002/(SICI)1097-0177(199606)206:2<219::AID-AJA11>3.0.CO;2-Y

[156] Barone MV, Henchcliffe C, Baralle FE, and Paolella G (1989). Cell type specific trans-acting factors are involved in alternative splicing of human fibronectin pre-mRNA. EMBO J. 8, 1079–1085. 2545440 10.1002/j.1460-2075.1989.tb03476.x PMC400917

[157] Lim LP, and Sharp PA (1998). Alternative splicing of the fibronectin EIIIB exon depends on specific TGCATG repeats. Mol. Cell Biol 18, 3900–3906. 9632774 10.1128/mcb.18.7.3900 PMC108974

[158] Huh GS, and Hynes RO (1994). Regulation of alternative pre-mRNA splicing by a novel repeated hexanucleotide element. Genes Dev. 8, 1561–1574. 7958840 10.1101/gad.8.13.1561

[159] Dominguez D, Freese P, Alexis MS, Su A, Hochman M, Palden T, Bazile C, Lambert NJ, Van Nostrand EL, Pratt GA, (2018). Sequence, Structure, and Context Preferences of Human RNA Binding Proteins. Mol. Cell 70, 854–867.e9. 29883606 10.1016/j.molcel.2018.05.001 PMC6062212

[160] Hensel JA, Heineman BD, Kimble AL, Jellison ER, Reese B, and Murphy PA (2021). Identification of splice regulators of fibronectin-EIIIA and EIIIB by direct measurement of exon usage in a flow-cytometry based CRISPR screen. Sci. Rep 11, 19835. 34615942 10.1038/s41598-021-99079-1 PMC8494765

[161] Murphy PA, Butty VL, Boutz PL, Begum S, Kimble AL, Sharp PA, Burge CB, and Hynes RO (2018). Alternative RNA splicing in the endothelium mediated in part by Rbfox2 regulates the arterial response to low flow. eLife 7, e29494. 29293084 10.7554/eLife.29494 PMC5771670

[162] Jangi M, Boutz PL, Paul P, and Sharp PA (2014). Rbfox2 controls autoregulation in RNA-binding protein networks. Genes Dev. 28, 637–651. 24637117 10.1101/gad.235770.113 PMC3967051

[163] Lopez-Mejia IC, De Toledo M, Della Seta F, Fafet P, Rebouissou C, Deleuze V, Blanchard JM, Jorgensen C, Tazi J, and Vignais ML (2013). Tissue-specific and SRSF1-dependent splicing of fibronectin, a matrix protein that controls host cell invasion. Mol. Biol. Cell 24, 3164–3176. 23966470 10.1091/mbc.E13-03-0142 PMC3806663

[164] Xiang J, Natarajan SK, Tremmel M, Ma D, Mocco J, Hopkins LN, Siddiqui AH, Levy EI, and Meng H (2011). Hemodynamic–Morpho-logic Discriminants for Intracranial Aneurysm Rupture. Stroke 42, 144–152. 21106956 10.1161/STROKEAHA.110.592923 PMC3021316

[165] Cicha I, Wörner A, Urschel K, Beronov K, Goppelt-Struebe M, Ver-hoeven E, Daniel WG, and Garlichs CD (2011). Carotid Plaque Vulnerability: A Positive Feedback Between Hemodynamic and Biochemical Mechanisms. Stroke 42, 3502–3510. 21998063 10.1161/STROKEAHA.111.627265

[166] Cheng C, Tempel D, van Haperen R, van der Baan A, Grosveld F, Daemen MJAP, Krams R, and de Crom R (2006). Atherosclerotic Lesion Size and Vulnerability Are Determined by Patterns of Fluid Shear Stress. Circulation 113, 2744–2753. 16754802 10.1161/CIRCULATIONAHA.105.590018

[167] Boussel L, Rayz V, McCulloch C, Martin A, Acevedo-Bolton G, Lawton M, Higashida R, Smith WS, Young WL, and Saloner D (2008). Aneurysm Growth Occurs at Region of Low Wall Shear Stress. Stroke 39, 2997–3002. 18688012 10.1161/STROKEAHA.108.521617 PMC2661849

[168] Argraves WS, Tanaka A, Smith EP, Twal WO, Argraves KM, Fan D, and Haudenschild CC (2009). Fibulin-1 and fibrinogen in human atherosclerotic lesions. Histochem. Cell Biol 132, 559–565. 19693531 10.1007/s00418-009-0628-7

[169] Bhosale SD, Moulder R, Venäläinen MS, Koskinen JS, Pitkänen N, Juonala MT, Kähönen MAP, Lehtimäki TJ, Viikari JSA, Elo LL, (2018). Serum Proteomic Profiling to Identify Biomarkers of Premature Carotid Atherosclerosis. Sci. Rep 8, 9209. 29907817 10.1038/s41598-018-27265-9 PMC6003912

[170] Zhao Q, Zhou H, Li X, and Xiao B (2019). The mechanosensitive Piezo1 channel: a three-bladed propeller-like structure and a lever-like mechanogating mechanism. FEBS J. 286, 2461–2470. 30500111 10.1111/febs.14711

[171] Miyamoto T, Mochizuki T, Nakagomi H, Kira S, Watanabe M, Takayama Y, Suzuki Y, Koizumi S, Takeda M, and Tominaga M (2014). Functional role for Piezo1 in stretch-evoked Ca2+ influx and ATP release in Urothelial cell cultures. J. Biol. Chem 289, 16565–16575. 24759099 10.1074/jbc.M113.528638 PMC4047422

[172] Lee W, Leddy HA, Chen Y, Lee SH, Zelenski NA, McNulty AL, Wu J, Beicker KN, Coles J, Zauscher S, (2014). Synergy between Piezo1 and Piezo2 channels confers high-strain mechanosensitivity to articular cartilage. Proc. Natl. Acad. Sci. USA 111, E5114–E5122. 25385580 10.1073/pnas.1414298111 PMC4250098

[173] Cahalan SM, Lukacs V, Ranade SS, Chien S, Bandell M, and Patapoutian A (2015). Piezo1 links mechanical forces to red blood cell volume. eLife 4, e07370. 26001274 10.7554/eLife.07370 PMC4456639

[174] Faucherre A, Kissa K, Nargeot J, Mangoni ME, and Jopling C (2014). Piezo1 plays a role in erythrocyte volume homeostasis. Haemato-logica 99, 70–75. 10.3324/haematol.2013.086090 PMC4007942 23872304

[175] Santana Nunez D, Malik AB, Lee Q, Ahn SJ, Coctecon-Murillo A, Lazarko D, Levitan I, Mehta D, and Komarova YA (2023). Piezo1 induces endothelial responses to shear stress via soluble adenylyl Cyclase-IP3R2 circuit. iScience 26, 106661. 37168565 10.1016/j.isci.2023.106661 PMC10164902

[176] Ranade SS, Woo SH, Dubin AE, Moshourab RA, Wetzel C, Petrus M, Mathur J, Bégay V, Coste B, Mainquist J, (2014). Piezo2 is the major transducer of mechanical forces for touch sensation in mice. Nature 516, 121–125. 25471886 10.1038/nature13980 PMC4380172

[177] Maksimovic S, Nakatani M, Baba Y, Nelson AM, Marshall KL, Wellnitz SA, Firozi P, Woo SH, Ranade S, Patapoutian A, and Lumpkin EA (2014). Epidermal Merkel cells are mechanosensory cells that tune mammalian touch receptors. Nature 509, 617–621. 24717432 10.1038/nature13250 PMC4097312

[178] Woo SH, Ranade S, Weyer AD, Dubin AE, Baba Y, Qiu Z, Petrus M, Miyamoto T, Reddy K, Lumpkin EA, (2014). Piezo2 is required for Merkel-cell mechanotransduction. Nature 509, 622–626. 24717433 10.1038/nature13251 PMC4039622

[179] Schulz K, Hazelton-Cavill P, Alornyo KK, Edenhofer I, Lindenmeyer M, Lohr C, Huber TB, Denholm B, and Koehler S (2024). Piezo activity levels need to be tightly regulated to maintain normal morphology and function in pericardial nephrocytes. Sci. Rep 14, 28254. 39548228 10.1038/s41598-024-79352-9 PMC11568303

[180] Geng J, Liu W, Zhou H, Zhang T, Wang L, Zhang M, Li Y, Shen B, Li X, and Xiao B (2020). A Plug-and-Latch Mechanism for Gating the Mechanosensitive Piezo Channel. Neuron 106, 438–451.e6. 32142647 10.1016/j.neuron.2020.02.010

[181] Szczot M, Pogorzala LA, Solinski HJ, Young L, Yee P, Le Pichon CE, Chesler AT, and Hoon MA (2017). Cell-Type-Specific Splicing of Piezo2 Regulates Mechanotransduction. Cell Rep. 21, 2760–2771. 29212024 10.1016/j.celrep.2017.11.035 PMC5741189

[182] Lewis AH, Cui AF, McDonald MF, and Grandl J (2017). Transduction of Repetitive Mechanical Stimuli by Piezo1 and Piezo2 Ion Channels. Cell Rep. 19, 2572–2585. 28636944 10.1016/j.celrep.2017.05.079 PMC5646378

[183] Heyman NS, Cowles CL, Barnett SD, Wu YY, Cullison C, Singer CA, Leblanc N, and Buxton IL (2013). TREK-1 currents in smooth muscle cells from pregnant human myometrium. Am J Physiol Cell Physiol 305, C632–42. 10.1152/ajpcell.00324.2012. 23804201 PMC3761174

[184] Cowles CL, Wu YY, Barnett SD, Lee MT, Burkin HR, and Buxton ILO (2015). Alternatively Spliced Human TREK-1 Variants Alter TREK-1 Channel Function and Localization1. Biol. Reprod 93, 122. 26400398 10.1095/biolreprod.115.129791 PMC4712007

[185] Bechard E, Bride J, Le Guennec JY, Brette F, and Demion M (2022). TREK-1 in the heart: Potential physiological and pathophysiological roles. Front. Physiol 13, 1095102. 10.3389/fphys.2022.1095102. 36620226 PMC9815770

[186] Alloui A, Zimmermann K, Mamet J, Duprat F, Noël J, Chemin J, Guy N, Blondeau N, Voilley N, Rubat-Coudert C, (2006). TREK-1, a K+ channel involved in polymodal pain perception. EMBO J. 25, 2368–2376. 16675954 10.1038/sj.emboj.7601116 PMC1478167

[187] Hervieu GJ, Cluderay JE, Gray CW, Green PJ, Ranson JL, Randall AD, and Meadows HJ (2001). Distribution and expression of TREK-1, a two-pore-domain potassium channel, in the adult rat CNS. Neuroscience 103, 899–919. 11301200 10.1016/s0306-4522(01)00030-6

[188] Wu YY, Singer CA, and Buxton ILO (2012). Variants of stretch-activated two-pore potassium channel TREK-1 associated with preterm labor in humans. Biol. Reprod 87, 96. 22811574 10.1095/biolreprod.112.099499 PMC3507547

[189] Fink M, Duprat F, Lesage F, Reyes R, Romey G, Heurteaux C, and Lazdunski M (1996). Cloning, functional expression and brain localization of a novel unconventional outward rectifier K+ channel. EMBO J. 15, 6854–6862. 9003761 PMC452511

[190] Bockenhauer D, Zilberberg N, and Goldstein SA (2001). KCNK2: reversible conversion of a hippocampal potassium leak into a voltage-dependent channel. Nat. Neurosci 4, 486–491. 11319556 10.1038/87434

[191] Xian Tao L, Dyachenko V, Zuzarte M, Putzke C, Preisig-Müller R, Isenberg G, and Daut J (2006). The stretch-activated potassium channel TREK-1 in rat cardiac ventricular muscle. Cardiovasc. Res 69, 86–97. 16248991 10.1016/j.cardiores.2005.08.018

[192] Rinné S, Renigunta V, Schlichthörl G, Zuzarte M, Bittner S, Meuth SG, Decher N, Daut J, and Preisig-Müller R (2014). A splice variant of the two-pore domain potassium channel TREK-1 with only one pore domain reduces the surface expression of full-length TREK-1 channels. Pflugers Arch. 466, 1559–1570. 24196565 10.1007/s00424-013-1384-z

[193] Veale EL, Rees KA, Mathie A, and Trapp S (2010). Dominant Negative Effects of a Non-conducting TREK1 Splice Variant Expressed in Brain. J. Biol. Chem 285, 29295–29304. 20605797 10.1074/jbc.M110.108423 PMC2937962

[194] Abraham DM, Lee TE, Watson LJ, Mao L, Chandok G, Wang HG, Frangakis S, Pitt GS, Shah SH, Wolf MJ, and Rockman HA (2018). The two-pore domain potassium channel TREK-1 mediates cardiac fibrosis and diastolic dysfunction. J. Clin. Investig 128, 4843–4855. 30153110 10.1172/JCI95945 PMC6205385

[195] Li Z-B, Zhang H-X, Li L-L, and Wang X-L (2005). Enhanced expressions of arachidonic acid-sensitive tandem-pore domain potassium channels in rat experimental acute cerebral ischemia. Biochem. Biophys. Res. Commun 327, 1163–1169. 15652517 10.1016/j.bbrc.2004.12.124

[196] Devilliers M, Busserolles J, Lolignier S, Deval E, Pereira V, Alloui A, Christin M, Mazet B, Delmas P, Noel J, (2013). Activation of TREK-1 by morphine results in analgesia without adverse side effects. Nat. Commun 4, 2941. 24346231 10.1038/ncomms3941

[197] Austen K, Ringer P, Mehlich A, Chrostek-Grashoff A, Kluger C, Klingner C, Sabass B, Zent R, Rief M, and Grashoff C (2015). Extracellular rigidity sensing by talin isoform-specific mechanical linkages. Nat. Cell Biol 17, 1597–1606. 26523364 10.1038/ncb3268 PMC4662888

[198] Huveneers S, Oldenburg J, Spanjaard E, van der Krogt G, Grigoriev I, Akhmanova A, Rehmann H, and de Rooij J (2012). Vinculin associates with endothelial VE-cadherin junctions to control force-dependent remodeling. J. Cell Biol 196, 641–652. 22391038 10.1083/jcb.201108120 PMC3307691

[199] del Rio A, Perez-Jimenez R, Liu R, Roca-Cusachs P, Fernandez JM, and Sheetz MP (2009). Stretching Single Talin Rod Molecules Activates Vinculin Binding. Science 323, 638–641. 19179532 10.1126/science.1162912 PMC9339221

[200] Grashoff C, Hoffman BD, Brenner MD, Zhou R, Parsons M, Yang MT, McLean MA, Sligar SG, Chen CS, Ha T, and Schwartz MA (2010). Measuring mechanical tension across vinculin reveals regulation of focal adhesion dynamics. Nature 466, 263–266. 20613844 10.1038/nature09198 PMC2901888

[201] Humphries JD, Wang P, Streuli C, Geiger B, Humphries MJ, and Ballestrem C (2007). Vinculin controls focal adhesion formation by direct interactions with talin and actin. J. Cell Biol 179, 1043–1057. 18056416 10.1083/jcb.200703036 PMC2099183

[202] Thompson PM, Tolbert CE, Shen K, Kota P, Palmer SM, Plevock KM, Orlova A, Galkin VE, Burridge K, Egelman EH, (2014). Identification of an Actin Binding Surface on Vinculin that Mediates Mechanical Cell and Focal Adhesion Properties. Structure 22, 697–706. 24685146 10.1016/j.str.2014.03.002 PMC4039106

[203] Rangarajan ES, Lee JH, Yogesha SD, and Izard T (2010). A Helix Replacement Mechanism Directs Metavinculin Functions. PLoS One 5, e10679. 20502710 10.1371/journal.pone.0010679 PMC2873289

[204] Byrne BJ, Kaczorowski YJ, Coutu MD, and Craig SW (1992). Chicken vinculin and meta-Vinculin are derived from a single gene by alternative splicing of a 207-base pair exon unique to meta-Vinculin. J. Biol. Chem 267, 12845–12850. 1618784

[205] Janssen MEW, Liu H, Volkmann N, and Hanein D (2012). The C-terminal tail domain of metavinculin, vinculin’s splice variant, severs actin filaments. J. Cell Biol 197, 585–593. 22613835 10.1083/jcb.201111046 PMC3365496

[206] Kim LY, Thompson PM, Lee HT, Pershad M, Campbell SL, and Alushin GM (2016). The Structural Basis of Actin Organization by Vinculin and Metavinculin. J. Mol. Biol 428, 10–25. 26493222 10.1016/j.jmb.2015.09.031 PMC4738167

[207] Oztug Durer ZA, McGillivary RM, Kang H, Elam WA, Vizcarra CL, Hanein D, De La Cruz EM, Reisler E, and Quinlan ME (2015). Meta-vinculin Tunes the Flexibility and the Architecture of Vinculin-Induced Bundles of Actin Filaments. J. Mol. Biol 427, 2782–2798. 26168869 10.1016/j.jmb.2015.07.005 PMC4540644

[208] Rüdiger M, Korneeva N, Schwienbacher C, Weiss EE, and Jockusch BM (1998). Differential actin organization by vinculin isoforms: Implications for cell type-specific microfilament anchorage. FEBS Lett. 431, 49–54. 9684863 10.1016/s0014-5793(98)00723-6

[209] Belkin AM, Ornatsky OI, Glukhova MA, and Koteliansky VE (1988). Immunolocalization of meta-vinculin in human smooth and cardiac muscles. J. Cell Biol 107, 545–553. 3138246 10.1083/jcb.107.2.545 PMC2115213

[210] Witt S, Zieseniss A, Fock U, Jockusch BM, and Illenberger S (2004). Comparative Biochemical Analysis Suggests That Vinculin and Metavinculin Cooperate in Muscular Adhesion Sites. J. Biol. Chem 279, 31533–31543. 15159399 10.1074/jbc.M314245200

[211] Olson TM, Illenberger S, Kishimoto NY, Huttelmaier S, Keating MT, and Jockusch BM (2002). Metavinculin Mutations Alter Actin Interaction in Dilated Cardiomyopathy. Circulation 105, 431–437. 11815424 10.1161/hc0402.102930

[212] Vasile VC, Will ML, Ommen SR, Edwards WD, Olson TM, and Ackerman MJ (2006). Identification of a metavinculin missense mutation, R975W, associated with both hypertrophic and dilated cardiomyopathy. Mol. Genet. Metab 87, 169–174. 16236538 10.1016/j.ymgme.2005.08.006

[213] Maeda M, Holder E, Lowes B, Valent S, and Bies RD (1997). Dilated Cardiomyopathy Associated With Deficiency of the Cytoskeletal Protein Metavinculin. Circulation 95, 17–20. 8994410 10.1161/01.cir.95.1.17

[214] Turner CE, and Burridge K (1989). Detection of metavinculin in human platelets using a modified talin overlay assay. Eur. J. Cell Biol 49, 202–206. 2503380

[215] Lee HT, Sharek L, O’Brien ET, Urbina FL, Gupton SL, Superfine R, Burridge K, and Campbell SL (2019). Vinculin and metavinculin exhibit distinct effects on focal adhesion properties, cell migration, and mechanotransduction. PLoS One 14, e0221962. 31483833 10.1371/journal.pone.0221962 PMC6726196

[216] Kanoldt V, Kluger C, Barz C, Schweizer AL, Ramanujam D, Wind-gasse L, Engelhardt S, Chrostek-Grashoff A, and Grashoff C (2020). Metavinculin modulates force transduction in cell adhesion sites. Nat. Commun 11, 6403. 33335089 10.1038/s41467-020-20125-z PMC7747745

[217] Krishnamoorthy GP, Glover AR, Untch BR, Sigcha-Coello N, Xu B, Vukel D, Liu Y, Tiedje V, Pineda JMB, Berman K, (2025). RBM10 loss promotes metastases by aberrant splicing of cytoskeletal and extracellular matrix mRNAs. J. Exp. Med 222, e20241029. 39992626 10.1084/jem.20241029 PMC11849553

[218] Caprara GA, Mecca AA, Wang Y, Ricci AJ, and Peng AW (2019). Hair Bundle Stimulation Mode Modifies Manifestations of Mechanotransduction Adaptation. J. Neurosci 39, 9098–9106. 31578232 10.1523/JNEUROSCI.1408-19.2019 PMC6855673

[219] Hudspeth AJ (2008). Making an Effort to Listen: Mechanical Amplification in the Ear. Neuron 59, 530–545. 18760690 10.1016/j.neuron.2008.07.012 PMC2724262

[220] Petit C, and Richardson GP (2009). Linking genes underlying deafness to hair-bundle development and function. Nat. Neurosci 12, 703–710. 19471269 10.1038/nn.2330 PMC3332156

[221] Kawashima Y, Géléoc GSG, Kurima K, Labay V, Lelli A, Asai Y, Makishima T, Wu DK, Della Santina CC, Holt JR, and Griffith AJ (2011). Mechanotransduction in mouse inner ear hair cells requires transmembrane channel-like genes. J. Clin. Investig 121, 4796–4809. 22105175 10.1172/JCI60405 PMC3223072

[222] Cunningham CL, and Müller U (2019). Molecular Structure of the Hair Cell Mechanoelectrical Transduction Complex. Cold Spring Harb. Perspect. Med 9, a033167. 10.1101/cshperspect.a033167 PMC6496331 30082452

[223] Ahmed ZM, Goodyear R, Riazuddin S, Lagziel A, Legan PK, Behra M, Burgess SM, Lilley KS, Wilcox ER, Riazuddin S, (2006). The tip-link antigen, a protein associated with the transduction complex of sensory hair cells, is protocadherin-15. J. Neurosci 26, 7022–7034. 16807332 10.1523/JNEUROSCI.1163-06.2006 PMC6673907

[224] Zhou Z, Yu X, Jiang B, Feng W, Tian Y, Liu Z, Wang J, and Huang P (2021). Alternative Splicing of Three Genes Encoding Mechanotransduction-Complex Proteins in Auditory Hair Cells. eNeuro 8, ENEURO.0381–20.2020. 10.1523/ENEURO.0381-20.2020 PMC7920537 33509951

[225] Pepermans E, Michel V, Goodyear R, Bonnet C, Abdi S, Dupont T, Gherbi S, Holder M, Makrelouf M, Hardelin JP, (2014). The CD 2 isoform of protocadherin-15 is an essential component of the tip-link complex in mature auditory hair cells. EMBO Mol. Med 6, 984–992. 24940003 10.15252/emmm.201403976 PMC4119359

[226] Corey DP, Akyuz N, and Holt JR (2019). Function and Dysfunction of TMC Channels in Inner Ear Hair Cells. Cold Spring Harb. Perspect. Med 9, a033506. 10.1101/cshperspect.a033506 PMC6450785 30291150

[227] Pan B, Akyuz N, Liu XP, Asai Y, Nist-Lund C, Kurima K, Derfler BH, György B, Limapichat W, Walujkar S, (2018). TMC1 Forms the Pore of Mechanosensory Transduction Channels in Vertebrate Inner Ear Hair Cells. Neuron 99, 736–753.e6. 30138589 10.1016/j.neuron.2018.07.033 PMC6360533

[228] Kurima K, Peters LM, Yang Y, Riazuddin S, Ahmed ZM, Naz S, Arnaud D, Drury S, Mo J, Makishima T, (2002). Dominant and recessive deafness caused by mutations of a novel gene, TMC1, required for cochlear hair-cell function. Nat. Genet 30, 277–284. 11850618 10.1038/ng842

[229] Steel KP, and Bock GR (1980). The nature of inherited deafness in deafness mice. Nature 288, 159–161. 7432512 10.1038/288159a0

[230] Deol MS, and Kocher W (1958). A new gene for deafness in the mouse. Heredity 12, 463–466.

[231] Yamaguchi S, Hamamura M, and Otsuguro KI (2020). Mechanosensitive Channel, Mouse Transmembrane Channel-Like Protein 1 (mTMC1) Is Translated from a Splice Variant mTmc1ex1 but Not from the Other Variant mTmc1ex2. Int. J. Mol. Sci 21, 6465. 32899784 10.3390/ijms21186465 PMC7554986

[232] Li Y, Liu H, Giffen KP, Chen L, Beisel KW, and He DZZ (2018). Transcriptomes of cochlear inner and outer hair cells from adult mice. Sci. Data 5, 180199. 30277483 10.1038/sdata.2018.199 PMC6167952

[233] Qiu X, Liang X, Llongueras JP, Cunningham C, and Müller U (2023). The tetraspan LHFPL5 is critical to establish maximal force sensitivity of the mechanotransduction channel of cochlear hair cells. Cell Rep. 42, 112245. 36917610 10.1016/j.celrep.2023.112245 PMC10337519

[234] Xiong W, Grillet N, Elledge HM, Wagner TFJ, Zhao B, Johnson KR, Kazmierczak P, and Müller U (2012). TMHS Is an Integral Component of the Mechanotransduction Machinery of Cochlear Hair Cells. Cell 151, 1283–1295. 23217710 10.1016/j.cell.2012.10.041 PMC3522178

[235] Longo-Guess CM, Gagnon LH, Fritzsch B, and Johnson KR (2007). Targeted knockout and lacZ reporter expression of the mouse Tmhs deafness gene and characterization of the hscy-2J mutation. Mamm. Genome 18, 646–656. 17876667 10.1007/s00335-007-9049-x PMC2613174

[236] Cunningham CL, Qiu X, Wu Z, Zhao B, Peng G, Kim YH, Lauer A, and Müller U (2020). TMIE Defines Pore and Gating Properties of the Mechanotransduction Channel of Mammalian Cochlear Hair Cells. Neuron 107, 126–143.e8. 32343945 10.1016/j.neuron.2020.03.033 PMC7351599

[237] Dionne G, Qiu X, Rapp M, Liang X, Zhao B, Peng G, Katsamba PS, Ahlsen G, Rubinstein R, Potter CS, (2018). Mechanotransduction by PCDH15 Relies on a Novel cis-Dimeric Architecture. Neuron 99, 480–492.e5. 30057206 10.1016/j.neuron.2018.07.006 PMC6168201

[238] Assad JA, Shepherd GM, and Corey DP (1991). Tip-link integrity and mechanical transduction in vertebrate hair cells. Neuron 7, 985–994. 1764247 10.1016/0896-6273(91)90343-x

[239] Webb SW, Grillet N, Andrade LR, Xiong W, Swarthout L, Della Santina CC, Kachar B, and Müller U (2011). Regulation of PCDH15 function in mechanosensory hair cells by alternative splicing of the cytoplasmic domain. Development 138, 1607–1617. 21427143 10.1242/dev.060061 PMC3062428

[240] Rouget-Quermalet V, Giustiniani J, Marie-Cardine A, Beaud G, Besnard F, Loyaux D, Ferrara P, Leroy K, Shimizu N, Gaulard P, (2006). Protocadherin 15 (PCDH15): a new secreted isoform and a potential marker for NK/T cell lymphomas. Oncogene 25, 2807–2811. 16369489 10.1038/sj.onc.1209301

[241] Wang Y, Zhang C, Peng W, Du H, Xi Y, and Xu Z (2023). RBM24 is required for mouse hair cell development through regulating pre-mRNA alternative splicing and mRNA stability. J. Cell. Physiol 238, 1095–1110. 36947695 10.1002/jcp.31003

[242] Merkin J, Russell C, Chen P, and Burge CB (2012). Evolutionary Dynamics of Gene and Isoform Regulation in Mammalian Tissues. Science 338, 1593–1599. 23258891 10.1126/science.1228186 PMC3568499

[243] Brinegar AE, Xia Z, Loehr JA, Li W, Rodney GG, and Cooper TA (2017). Extensive alternative splicing transitions during postnatal skeletal muscle development are required for calcium handling functions. eLife 6, e27192. 28826478 10.7554/eLife.27192 PMC5577920

[244] Fugier C, Klein AF, Hammer C, Vassilopoulos S, Ivarsson Y, Toussaint A, Tosch V, Vignaud A, Ferry A, Messaddeq N, (2011). Misregulated alternative splicing of BIN1 is associated with T tubule alterations and muscle weakness in myotonic dystrophy. Nat. Med 17, 720–725. 21623381 10.1038/nm.2374

[245] Giudice J, Loehr JA, Rodney GG, and Cooper TA (2016). Alternative Splicing of Four Trafficking Genes Regulates Myofiber Structure and Skeletal Muscle Physiology. Cell Rep. 17, 1923–1933. 27851958 10.1016/j.celrep.2016.10.072 PMC5140099

[246] Moulay G, Lainé J, Lemaître M, Nakamori M, Nishino I, Caillol G, Mamchaoui K, Julien L, Dingli F, Loew D, (2020). Alternative splicing of clathrin heavy chain contributes to the switch from coated pits to plaques. J. Cell Biol 219, e201912061. 32642759 10.1083/jcb.201912061 PMC7480091

[247] Yang J, Hung LH, Licht T, Kostin S, Looso M, Khrameeva E, Bindereif A, Schneider A, and Braun T (2014). RBM24 Is a Major Regulator of Muscle-Specific Alternative Splicing. Dev. Cell 31, 87–99. 25313962 10.1016/j.devcel.2014.08.025

[248] Creemers EE, Bawazeer A, Ugalde AP, van Deutekom HWM, van der Made I, de Groot NE, Adriaens ME, Cook SA, Bezzina CR, Hubner N, (2016). Genome-Wide polyadenylation maps reveal dynamic mRNA 3^′^-End formation in the failing human heart. Circ. Res 118, 433–438. 26671978 10.1161/CIRCRESAHA.115.307082

[249] Soetanto R, Hynes CJ, Patel HR, Humphreys DT, Evers M, Duan G, Parker BJ, Archer SK, Clancy JL, Graham RM, (2016). Role of miRNAs and alternative mRNA 3^′^-end cleavage and polyadenylation of their mRNA targets in cardiomyocyte hypertrophy. Biochim. Biophys. Acta 1859, 744–756. 27032571 10.1016/j.bbagrm.2016.03.010

[250] Mattias F, Tsoy O, Hammer E, Gress A, Simm S, Lio CT, Ameling S, Amann K, Dreher L, Wenzel U, (2025). Alternative Splicing in Mechanically Stretched Podocytes as a Model of Glomerular Hypertension. J. Am. Soc. Nephrol 10.1681/ASN.0000000706. PMC12416947 40418580

[251] Endlich N, Kress KR, Reiser J, Uttenweiler D, Kriz W, Mundel P, and Endlich K (2001). Podocytes respond to mechanical stress in vitro. J. Am. Soc. Nephrol 12, 413–422. 11181788 10.1681/ASN.V123413

[252] Friedrich C, Endlich N, Kriz W, and Endlich K (2006). Podocytes are sensitive to fluid shear stress in vitro. Am. J. Physiol. Renal Physiol 291, F856–F865. 16684926 10.1152/ajprenal.00196.2005

[253] Endlich K, Kliewe F, and Endlich N (2017). Stressed podocytes-mechanical forces, sensors, signaling and response. Pflugers Arch. 469, 937–949. 28687864 10.1007/s00424-017-2025-8

[254] Shi Y (2012). Alternative polyadenylation: New insights from global analyses. Rna 18, 2105–2117. 23097429 10.1261/rna.035899.112 PMC3504663

[255] Li L, Huang KL, Gao Y, Cui Y, Wang G, Elrod ND, Li Y, Chen YE, Ji P, Peng F, (2021). An atlas of alternative polyadenylation quantitative trait loci contributing to complex trait and disease heritability. Nat. Genet 53, 994–1005. 33986536 10.1038/s41588-021-00864-5

[256] Mariella E, Marotta F, Grassi E, Gilotto S, and Provero P (2019). The Length of the Expressed 3′ UTR Is an Intermediate Molecular Phenotype Linking Genetic Variants to Complex Diseases. Front. Genet 10, 714. 31475030 10.3389/fgene.2019.00714 PMC6707137

[257] Mittleman BE, Pott S, Warland S, Zeng T, Mu Z, Kaur M, Gilad Y, and Li Y (2020). Alternative polyadenylation mediates genetic regulation of gene expression. eLife 9, e57492. 32584258 10.7554/eLife.57492 PMC7338057

[258] Sadek J, Omer A, Hall D, Ashour K, and Gallouzi IE (2019). Alternative polyadenylation and the stress response. WIREs RNA 10, e1540. 31050180 10.1002/wrna.1540

[259] Hollerer I, Curk T, Haase B, Benes V, Hauer C, Neu-Yilik G, Bhuvanagiri M, Hentze MW, and Kulozik AE (2016). The differential expression of alternatively polyadenylated transcripts is a common stress-induced response mechanism that modulates mammalian mRNA expression in a quantitative and qualitative fashion. RNA 22, 1441–1453. 27407180 10.1261/rna.055657.115 PMC4986898

[260] Zheng D, Wang R, Ding Q, Wang T, Xie B, Wei L, Zhong Z, and Tian B (2018). Cellular stress alters 3^′^UTR landscape through alternative polyadenylation and isoform-specific degradation. Nat. Commun 9, 2268. 29891946 10.1038/s41467-018-04730-7 PMC5995920

[261] Weber M, Hagedorn CH, Harrison DG, and Searles CD (2005). Laminar shear stress and 3’ polyadenylation of eNOS mRNA. Circ. Res 96, 1161–1168. 15905462 10.1161/01.RES.0000170651.72198.fa

[262] Lyon RC, Zanella F, Omens JH, and Sheikh F (2015). Mechanotransduction in Cardiac Hypertrophy and Failure. Circ. Res 116, 1462–1476. 25858069 10.1161/CIRCRESAHA.116.304937 PMC4394185

[263] Lammerding J, Kamm RD, and Lee RT (2004). Mechanotransduction in cardiac myocytes. Ann. N. Y. Acad. Sci 1015, 53–70. 15201149 10.1196/annals.1302.005

[264] Gruber AJ, and Zavolan M (2019). Alternative cleavage and polyadenylation in health and disease. Nat. Rev. Genet 20, 599–614. 31267064 10.1038/s41576-019-0145-z

[265] Zhang Y, and Li Z (2021). RNA binding proteins: Linking mechanotransduction and tumor metastasis. Cancer Lett. 496, 30–40. 33007411 10.1016/j.canlet.2020.09.020

[266] Seufert L, Benzing T, Ignarski M, and Müller RU (2022). RNA-binding proteins and their role in kidney disease. Nat. Rev. Nephrol 18, 153–170. 10.1038/s41581-021-00497-1. 34732838

[267] Hastings ML, and Krainer AR (2023). RNA therapeutics. RNA 29, 393–395. 10.1261/rna.079626.123. 36928165 PMC10019368

[268] Sachse M, Tual-Chalot S, Ciliberti G, Amponsah-Offeh M, Stamate-lopoulos K, Gatsiou A, and Stellos K (2023). RNA-binding proteins in vascular inflammation and atherosclerosis. Atherosclerosis 374, 55–73. 10.1016/j.atherosclerosis.2023.01.008. 36759270

[269] Takakusa H, Iwazaki N, Nishikawa M, Yoshida T, Obika S, and Inoue T (2023). Drug Metabolism and Pharmacokinetics of Antisense Oligonucleotide Therapeutics: Typical Profiles, Evaluation Approaches, and Points to Consider Compared with Small Molecule Drugs. Nucleic Acid Ther. 33, 83–94. 10.1089/nat.2022.0054. 36735616 PMC10066781

[270] Black AJ, Gamarra JR, and Giudice J (2019). More than a messenger: Alternative splicing as a therapeutic target. Biochim. Biophys. Acta. Gene Regul. Mech 1862, 194395. 31271898 10.1016/j.bbagrm.2019.06.006 PMC6875611

